# Effect of Parkinson’s disease and related medications on the composition of the fecal bacterial microbiota

**DOI:** 10.1038/s41531-019-0100-x

**Published:** 2019-11-29

**Authors:** Severin Weis, Andreas Schwiertz, Marcus M. Unger, Anouck Becker, Klaus Faßbender, Stefan Ratering, Matthias Kohl, Sylvia Schnell, Karl-Herbert Schäfer, Markus Egert

**Affiliations:** 1 Faculty of Medical and Life Sciences, Institute of Precision Medicine, Microbiology and Hygiene Group, Furtwangen University, Villingen-Schwenningen, Germany; 2 MVZ Institute of Microecology, Herborn, Germany; 3 Department of Neurology, Saarland University, Homburg, Germany; 4 Institute of Applied Microbiology, Justus-Liebig-University, Giessen, Germany; 5 Faculty of Medical and Life Sciences, Institute of Precision Medicine, Group for Statistics in Biology and Medicine, Furtwangen University, Villingen-Schwenningen, Germany; 6 Department of Biotechnology, ENS Working Group, University of Applied Sciences Kaiserslautern, Zweibrücken, Germany

**Keywords:** Constipation, Parkinson's disease

## Abstract

Parkinson’s disease (PD) is one of the most common neurodegenerative disorders. PD patients suffer from gastrointestinal dysfunctions and alterations of the autonomous nervous system, especially its part in the gut wall, i.e., the enteric nervous system (ENS). Such alterations and functional gastrointestinal deficits often occur years before the classical clinical symptoms of PD appear. Until now, only little is known about PD-associated changes in gut microbiota composition and their potential implication in PD development. In order to increase knowledge in this field, fecal samples of 34 PD patients and 25 healthy, age-matched control persons were investigated. Here, the V4 and V5 hypervariable region of bacterial 16S rRNA genes was PCR-amplified and sequenced using an Ion Torrent PGM platform. Within the PD group, we observed a relative decrease in bacterial taxa which are linked to health-promoting, anti-inflammatory, neuroprotective or other beneficial effects on the epithelial barrier, such as *Faecalibacterium* and *Fusicatenibacter*. Both taxa were lowered in PD patients with elevated levels of the fecal inflammation marker calprotectin. In addition, we observed an increase in shares of the *Clostridiales* family XI and their affiliated members in these samples. Finally, we found that the relative abundances of the bacterial genera *Peptoniphilus, Finegoldia, Faecalibacterium Fusicatenibacter, Anaerococcus, Bifidobacterium, Enterococcus*, and *Ruminococcus* were significantly influenced by medication with L-dopa and entacapone, respectively. Our data confirm previously reported effects of COMT inhibitors on the fecal microbiota of PD patients and suggest a possible effect of L-dopa medication on the relative abundance of several bacterial genera.

## Introduction

Parkinson’s disease (PD) is one of the most common neurodegenerative disorders. So far, there is no causal treatment for PD that is able to halt the neurodegenerative process.^1^ Besides motor and cognitive symptoms, most PD patients suffer from gastrointestinal (GI) symptoms, such as constipation or prolonged intestinal transit time.^2–5^ These symptoms can occur several years ahead of classical motor symptoms, which gives rise to the hypothesis that the enteric nervous system (ENS) becomes compromised before the central nervous system.^6–8^ These observations support the hypothesis that PD may begin in the GI tract.^9^

The ENS is a complex of several networks of neurons, glial cells, and interconnecting fibers within the mammal GI tract. The ENS communicates with the brain bidirectionally via the vagus nerve as the hard-wired section of the so called brain-gut axis.^10^ Experimental data suggest that neurons and fibers from the ENS and vagus nerve provide a neuronal chain, that allow pathological peptides to travel between the gut and the brain in a prion-like way and modulate the course of neurological diseases.^11–13^

Recent studies indicate, that the pathological process of PD alongside the gut-brain-axis might be modulated or even initiated by the gut microbiota.^14,15^ Indicators are fecal markers of gut inflammation and permeability, which are increased in PD.^16^ Furthermore, bacterial metabolites, which may have an influence on the ENS, differ between PD patients and healthy controls.^16,17^ Clearly, the cause-effect relationship between the intestinal microbiota composition and its metabolic capacity on the one hand and PD pathogenesis on the other hand are still obscure. Constipation and reduced gut motility in PD may be important triggers to alter the microbiota composition.^18,19^

In addition, previous studies also suggested that some PD medication may alter the microbiota composition.^20–22^ L-dopa (L-3,4-Dihydroxyphenylalanine, levodopa) application targets the striatal dopamine deficiency in PD patients and may stimulate the dopamine transporter on the terminal nerve.^23,24^ Long term therapy with L-dopa is known to induce side effects like increased inflammation and oxidative stress.^23^ Catechol-O-methyltransferases (COMT) are able to methylate L-dopa rendering it ineffective.^25,26^ COMT are able to methylate a wide range of catechols and thereby eliminate biologically active or toxic molecules^27^ including L-dopa.^25,26,28^ Entacapone is a COMT inhibitor^28^ preventing the degradation and increasing the plasma availability of L-dopa.^28,29^ To the best of our knowledge, it is still unknown in which mechanistic way these drugs may alter gut microbiota composition and/or functionality.

Increasing evidence indicates a difference in fecal microbiota composition between PD and healthy controls, irrespective of the applied method.^17,22,30–32^ The methods used so far were quantitative real-time PCR (qPCR) to detect highly abundant bacterial taxa,^17^ pyrosequencing of the V4 variable region of the 16S rRNA gene^14^ and Illumina MiSeq sequencing of the V1 and V2 region^32^ or V4 region.^22^ Other studies addressed the microbial and viral gut metagenome in the early stage of PD^33^ in L-dopa naïve patients with shotgun sequencing methods.^34^ Correlations between a medication with entacapone and the (relative) abundances of distinct bacterial taxa were previously reported,^17,22,30^ suggesting PD medication as an important influencing factor for the PD microbiota. However, to the best of our knowledge no study reported medication with L-dopa as an influencing factor so far.

In this study, we used next generation sequencing to screen for difference in fecal microbiota composition between PD patients and matched controls. We analyzed the same set of samples previously detailed by Unger et al.,^17^ albeit with Ion Torrent-based next generation sequencing of the V4 and V5 region of the bacterial 16S rRNA gene instead of qPCR. While qPCR is suitable for the quantitative determination of distinct bacterial taxa, it is also limited to known sequence types. We believe that monitoring differences in fecal microbiota composition in PD patients compared to suitable controls is a first step to elucidate whether the gut microbiota might play any functional role in PD pathogenesis. Clearly, such differences might also depend on factors such as the type of PD medication. In the long run, microorganisms which differ significantly between healthy persons and PD patients (independent on factors such as medication) might play an indicative role in early PD diagnosis. This may increase our knowledge on the aetiopathogenesis of PD.

## Results

### Sequencing and bioinformatics

Four combined sequencing datasets yielded 11,752,187 partial bacterial 16S rRNA gene sequences with a mean of 199,190 sequences per sample (min: 70,225; max: 394,784 sequences). Following exclusion of sequences that were present in less than 10% of all samples, 12,935 OTUs affiliated with 192 genera, 55 families, 28 orders, 17 classes, and 8 phyla were identified.

Searching for interdependencies among the sample attributes, CramérV contingency coefficients did not show strong association (cV > 0.7)^35^ and no significant *Χ*² *p*-values were detected between most of the sample attributes. Only, L-dopa medication showed a significance in the *Χ*²-test (*p* = 0.048) and a medium strong association with the Hoehn–Yahr stage (cV = 0.53). According to the Spearman-*Ρ*, the Hoehn–Yahr stage was significantly positively correlated with disease duration (*p* = 0.0016; *Ρ* = 0.5183). The numbers of samples per investigated patient or control subgroup are summarized in Table 1. Important metadata are detailed in the Supplementary Table 1.

**Table 1:** Samples sizes in the investigated cohort.

	PD	Ctrl
Whole group	34	25
Sex		
Male (m)	23	11
Female (f)	11	14
Smoker		
Yes	2	7
No	32	18
Appendectomy		
Yes	15	NA
No	19	NA
Family history for neurodegenerative disorders		
Yes	8	NA
No	26	NA
Phenotype		
Tremor dominant (T)	6	–
Hypokinetic-rigid (HR)	15	–
Equivalent (E)	13	–
Hoehn-Yahr Stage		
1–2.5	18	–
3–4	16	–
Calprotectin		
Positive	14	3
Negative	20	22
Constipation		
Yes	7	2
No	27	23
Other GI symptoms		
Yes	8	-
No	26	25
Entacapone treatment		
Yes	11	–
No	23	25
L-dopa treatment		
Yes	24	–
No	10	25

Shown are the sample sizes in the examined sub-groups. Sex is divided into female (f) and male (m), family history indicates whether there was a family history for neurodegenerative disorders or not, and phenotype was defined as hypokinetic-rigid (HR), tremor dominant (T), and equivalent (E). Calprotectin was regarded as positive, when concentrations exceeded 50 µg/g. Constipation was defined as less than three bowel movements a week or bowel movements that were hard, dry, small, painful or difficult to pass. Other GI symptoms were pyrosis (*n* = 5), intermittent abdominal pain (*n* = 1), flatulence (*n* = 1), and occasional nausea (*n* = 1)

### Structural diversity measures

**Fig. 1:**
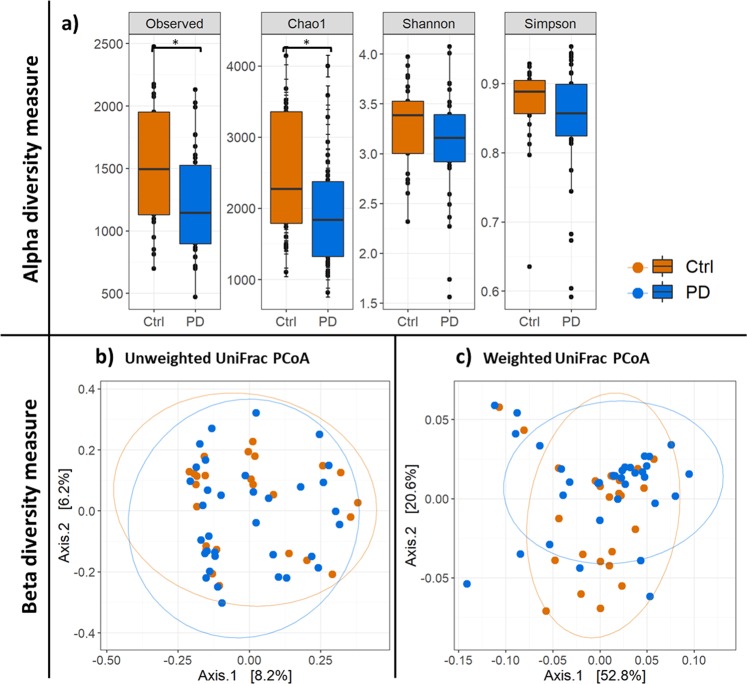
Alpha and beta diversity plots to visualize the difference in microbiota structure between PD and control group. Shown are alpha diversity measures with the most common indices (**a**) and PCoA plots showing the beta diversity with unweighted (**b**) and weighted (**c**) UniFrac measures. Blue: PD samples, orange: controls. Box plots (**a**) show median, as well as lower and upper quartiles. Each dot represents an individual sample. Whiskers represent minimum and maximum spread. PCoA plots show dimensions with the highest differences, and normal confidence ellipsoids for the sample sets.

Alpha diversity indices (Fig. 1a) revealed a significant decrease in bacterial diversity in PD patients compared to the control (Ctrl) regarding observed species (*p*_Observed = _0.032) and estimated species (*p*_Chao1_ = 0.032). Shannon and Simpson metrics for alpha diversity did not show significant differences between PD and control (*p*_Shannon_ = 0.197, *p*_Simpson_ = 0.197).

Non-parametric multivariate analysis of variance (ADONIS), calculated for the beta diversity of PD microbiota and the control microbiota (Fig. 1b + c), revealed that neither the weighted (*p*_weighted-UniFrac_ = 0.249) nor the unweighted (*p*_unweighted UniFrac_ = 0.226) UniFrac measure were significantly different between the PD and the control group.

### Differences in microbiota composition

One bacterial family and three genera were found to be significantly different in relative abundance between PD and controls (*p* < 0.05). The *Clostridiales* family XI and the genus *Peptoniphilus* were observed at higher relative abundances in the PD dataset. The genera *Faecalibacterium* and *Fusicatenibacter* decreased relatively in PD (Table 2).

**Table 2:** Genera differing significantly within the tested attribute family.

		Peptoniphilus	Finegoldia	Anaerococcus	Eubacterium brachy group	Faecalibacterium	Fusicatenibacter	Ruminococcus gauvreauii group	Sellimonas	Bifidobacterium	Streptococcus	Enterococcus	Dielma
Disease													
Ctrl/PD	P(FDR) A/B	<0.001 0.00				0.019 3.06	0.035 12.56						
Sex													
Ctrl-f/Crtl-m	P(FDR) A/B									0.005 12.60			
PD-f/PD-m	P(FDR) A/B												
Ctrl-f/PD-f	P(FDR) A/B	<0.001 0.00				0.009 9.89							
Ctrl-m/PD-m	P(FDR) A/B												
Calprotectin													
Ctrl−/PD−	P(FDR) A/B	0.0192 0.00											
Ctrl−/PD+	P(FDR) A/B	<0.001 0.00	<0.001 0.33			<0.001 31.20	0.004 62.00						
Ctrl−/Ctrl+	P(FDR) A/B					0.048 8.50					0.048 0.61		
L-dopa													
Ctrl−/PD−	P(FDR) A/B												
Ctrl−/PD+	P(FDR) A/B	<0.001 0.00	0.019 0.00			<0.001 04.69		0.038 2.06					
Ctrl−/Ctrl+	P(FDR) A/B												
Entacapone													
Ctrl−/PD−	P(FDR) A/B	0.019 0.00											
Ctrl−/PD+	P(FDR) A/B	<0.001 0.00		0.025 0.20	0.025 0.75	0.025 10.25		0.03 51.00	0.031 0.09	0.009 0.29		0.025 0.90	
Ctrl−/Ctrl+	P(FDR) A/B									0.01 0.26			<0.001 0.03
Phenotype													
Ctrl/E	P(FDR) A/B												
Ctrl/HR	P(FDR) A/B	<0.001 0.00				<0.001 16.65							
Ctrl/T	P(FDR) A/B												
Ctrl/E	P(FDR) A/B												
E/HR	P(FDR) A/B												
E/T	P(FDR) A/B												
HR/T	P(FDR) A/B												
Hoehn Yahr stage													
Ctrl/HY 1-2.5	P(FDR) A/B	<0.001 0.00				0.029 7.61							
Ctrl/HY 3-4	P(FDR) A/B	<0.001 0.00											
HY 1-2.5/HY3-4	P(FDR) A/B												

Shown are the *p*-values for genera that differed significantly between the tested attributes. The quotient of relative abundances for each genus (A/B) indicates whether the genus relatively increased (>1) or decreased (<1) in the second attribute when compared to the first attribute. All *p*-values are based on false discovery correction for multiple testing (FDR) calculated within the attribute family. Compared were the control (Ctrl) and the Parkinson (PD) samples, and the attributes female (f) and male (m) in the sex attribute family, positive (+) and negative (−) detection of calprotectin in the fecal samples and medication (+) or no medication (−) with entacapone or L-dopa. Also shown are the PD phenotypes hypokinetic-rigid (HR), tremordominant (T) and equivalent (E) and the Hoehn Yahr stages HY 1-4

In the control group, the family *Bifidobacteriaceae* and the genus *Bifidobacterium* were relatively increased within the female controls in comparison to the male controls. In the PD group, no taxon was found to be significantly different in relative abundance between male and female patients. In the female samples, a significant increase in the relative abundance of the *Clostridiales* family XI and the genus *Peptoniphilus* was observed. Furthermore, a decrease in abundance for *Faecalibacterium* in PD was observed. Within the male samples with PD, the family of *Bifidobacteriaceae* was significantly increased in relative abundance.

PD patients with elevated levels of fecal calprotectin^16,17^ showed a significant increase in the relative abundance of the *Clostridiales* family XI, the genera *Peptoniphilus* and *Finegoldia*, and significantly decreased relative abundances for *Faecalibacterium* and *Fusicatenibacter* when compared to calprotectin negative controls. PD patients with normal calprotectin levels showed an increased relative abundance of *Peptoniphilus* in comparison to controls. In addition, there was an increase in relative abundance for *Faecalibacterium* and decreased relative abundance for the genus *Streptococcus* and the family *Streptococcaceae* when compared to calprotectin positive PD patients.

PD patients treated with L-dopa showed significantly higher relative abundances of *Enterococcaceae*, *Clostridiales* family XI, and the genera *Peptoniphilus* and *Finegoldia*, while *Faecalibacterium* and the *Ruminococcus gauvreauii* group were significantly decreased when compared to the controls. However, there were no significant differences on genus or family levels between PD patients who were not treated with L-dopa and the control group or the PD patients treated with L-dopa.

PD patients treated with entacapone showed significantly higher relative abundances of the families *Enterococcaceae, Bifidobacteriaceae*, and the *Clostridiales* family XI, as well as of the genera *Peptoniphilus, Anaerococcus*, the *Eubacterium brachy* group, *Sellimonas, Bifidobacterium*, and *Enterococcus*, when compared to the controls. However, the *Ruminococcus gauvreauii* group and the genus *Faecalibacterium* were significantly decreased in this relation.

PD patients with a hypokinetic-rigid (HR) phenotype showed a significantly increased relative abundance of *Peptoniphilus* and decreased relative abundance of *Faecalibacterium* compared to the control group, while PD patients with a tremor dominant (T) phenotype showed a significant decrease in the relative abundance of the *Ruminococcaceae* family.

Finally, PD patients were grouped according to their Hoehn–Yahr (HY) stage. The first group contained all patients with a HY stage of 1 to 2.5, and the second all patients with a HY stage of 3 to 4. PD patients of the first group show a significantly increased relative abundance of *Peptoniphilus* and *Faecalibacterium* when compared to the control group. PD patients of the second group only showed a significant increase in the relative abundance of *Peptoniphilus*. When comparing the two groups of PD patients to each other, no significant difference were observed.

### Functional diversity measures

In contrast to the alpha diversity indices calculated for the relative abundances of bacterial genera, alpha diversity measures of predicted pathway abundances using PICRUSt revealed a significant increase in alpha diversity in PD patients compared to the control regarding observed (*p*_Observed_ = 0.0435) and estimated (*p*_Chao1_ = 0.0496) metrics. Shannon and Simpson metrics did not show significant differences between PD and the control group.

PD patients treated with entacapone also showed a significantly higher diversity of predicted pathways according to observed (*p*_Observed_ = 0.0435) and estimated (*p*_Chao1_ = 0.0496) metrics when compared to the control group. When compared to patients that were not treated with entacapone, the diversity of predicted pathways was significantly higher in patients that were treated with entacapone according to observed (*p*_Observed_ = 0.0351) and Simpson (*p*_Simpson_ = 0.0315) metrics.

PD patients treated with L-dopa showed a borderline significance (*p*_Observed_ = 0.05) for the abundance of predicted pathways when compared to the control group. PD patients with a Hoehn–Yahr stage of three to four showed a significant increased alpha diversity (*p*_Observed_ = 0.0344) for the abundance of predicted pathways when compared to the control group.

Bray–Curtis analyses, calculated for beta diversity of the predicted pathways, revealed a significant difference between the PD patients treated with entacapone when compared to the controls and patients that were not treated with entacapone. Calculated *p*-values for the alpha and beta diversity of predicted pathways are detailed in the Supplementary Table 2. Pathways differing significantly between PD and controls and under L-dopa or entacapone treatment are displayed in the Supplementary Tables 3–5.

## Discussion

Besides classical motor symptoms, PD patients frequently show gastrointestinal dysfunctions including impaired gastric emptying, constipation, and defecatory dysfunction.^36^ In addition, it is discussed that constipation in PD might partly be dependent on changes of the intestinal microbiota composition and their metabolic products.^37^ The gut microbiota is influenced by diet, medication, the immune system, intestinal transit time, and other factors. Clearly, a basic understanding of compositional changes is needed to better understand, how the gut microbiota might influence onset and progression of PD, or is in turn influenced by the disease itself. Another point of interest is to understand how medication and severity of the illness impact the gut microbiota. Only a few publications addressed such changes of the intestinal microbiota in PD patients, applying different methods such as qPCR,^17^ pyrosequencing,^14,30^ and Illumina MiSeq sequencing of different regions of the 16S rRNA gene.^22,32^

Our data indicate a significantly decreased richness of the microbiota (observed and chao1) (Fig. 1a). This is in contrast with former studies, where no significant differences in microbial diversity measures between PD and controls were reported.^30,32^ However, in agreement with Hopfner and collegaues^32^ but in contrast to Scheperjans and colleagues,^30^ the beta diversity measures did not show significant difference between PD and control samples for the unweighted and weighted unifrac metric (Fig. 1b+c). This hints to a very similar composition of the bacterial microbiota of PD patients and the healthy control group. Therefore, our expectation was to find significant differences in relative abundance only for a few bacterial taxa.

Calprotecin is a fecal marker for inflammation and has been shown to be elevated in PD patients compared to control persons.^16^ In our study, the genera *Peptoniphilus* and *Finegoldia* showed a relative increase in abundance within the group of calprotectin positive PD patients. Both genera were reported as opportunistic pathogens and being affiliated with polymicrobial infections and inflammation.^38–40^ In contrast, the genera *Faecalibacterium* and *Fusicatenibacter* showed a relative decrease in abundance in the group of calprotectin positive PD patients. *Faecalibacterium* has been reported as anti-inflammatory and health promoting, while the administration of *Fusicatenibacter* was reported to improve murine colitis. Both genera were reported to be decreased in inflammatory bowel disease and ulcerative colitis.^41–45^ A link between intestinal inflammation as environmental factor and PD was previously reported.^16,46^ To the best of our knowledge, an association between calprotectin and the gut microbiota in PD has not been reported yet. Since none of the subjects reported a history of acute or chronic gastrointestinal disorders, the increase of the inflammation marker calprotectin might be an indicator of changes within the intestinal microbiota in PD that is associated with an asymptomatic, low-grade inflammation.

L-dopa represents the most potent dopamine replacement agent to treat PD.^47^ However, gut microorganisms are able to degrade L-dopa even in the presence of decarboxylase inhibitors, thereby reducing its effectiveness.^20,48,49^ We found *Peptoniphilus* and *Finegoldia* to be relatively increased in PD patients treated with L-dopa, but not in PD patients that were treated with other dopaminergic drugs. Both genera are capable of peptone and amino acid fermentation^50,51^ and might therefore play a role in the degradation of L-dopa. Previous studies found a borderline significant influence of L-dopa on the total gut microbiome or discussed the treatment with L-dopa as a possible influencing factor.^14,22,32^ Furthermore, a recent study showed the important role of microbial metabolism in drug availability and degradation in the gut.^20^ However, as far as we know, no study specifically addressed changes in microbial community composition in PD related to L-dopa medication in detail. Since several bacteria are capable of metabolizing this drug, further investigations might directly target bacteria involved in this degradation process.

Entacapone is the most frequently used COMT inhibitor and has been shown to enhance the potency of L-dopa.^52^ When compared to the control group, patients treated with this drug showed the highest number of families and genera differing significantly in relative abundance. Only *Peptoniphilus* showed a difference in relative abundance also for the group of PD patients that received no entacapone treatment, when compared to the control. For COMT inhibitors gastrointestinal side effect such as diarrhea have been reported, which may lead to changes in gut microbiota composition.^52,53^ Several studies reported a possible link of COMT inhibitors to difference of abundance of some taxa.^17,22,30^ Especially an influence of entacapone on the abundance of *Faecalibacterium, Bifidobacterium, Lachnospiraceae, Blautia*, and *Enterobacteriaceae* were previously reported.^17,22,30^ Hill-Burns and colleagues reported the associations with PD and PD medication at OTU, genus, and family level to be robust.^22^ We were able to confirm a previously reported influence of entacapone on the relative abundances of *Faecalibacterium* and *Bifidobacterium*.^17,22^ In addition, we could show that *Anaerococcus*, the *Eubacterium brachy* group, *Sellimonas*, and *Enterococcus* increased significantly in relative abundance only in PD patients treated with entacapone.

*Faecalibacterium* showed a relative reduction in abundance in the PD samples compared to the controls, which corroborates several previous studies.^14,17,30^ Notably, in the study by Unger and colleagues,^17^ the identical set of samples was investigated, albeit using qPCR. For *Faecalibacterium*, health promoting and anti-inflammatory effects have been reported.^43–45,54^ The decreased relative abundance of this genus in conjunction with entacapone treatment, as seen in our study, is compatible with previously published findings.^14,17^ The potential anti-inflammatory effect of *Faecalibacterium* matches our observation of a significantly decreased relative abundance in PD patients who showed elevated levels of calprotectin, a fecal marker for gut inflammation, but not in those patients who showed normal values. *Faecalibacterium* was also found to be reduced in relative abundance in PD patients who were on L-dopa treatment, but not in patients who were on other dopaminergic treatments. Recent studies suggested L-dopa to induce inflammatory responses.^55^ Hence, the influence of L-dopa on anti-inflammatory bacterial genera like *Faecalibacterium* should be examined in more detail. Given that *Faecalibacterium* is one of the most important butyrate producers, with butyrate being the main energy source of the intestinal epithelium, a decrease of this genus might lead to lower butyrate levels^17^ and thus to an impairment of the gastrointestinal mucus layer,^56^ rendering the enteric nervous system more susceptible to intraluminal pathogens and inflammation.

*Peptoniphilus* is affiliated with the *Clostridiales* family XI and was significantly increased in relative abundance in PD patients. This genus belongs to the normal gut microbiota of humans, as well as the skin, mouth, and upper respiratory tract. Nevertheless, it has been observed in the context of polymicrobial infections, inflammation of the upper respiratory tract, septic arthritis, and diabetes mellitus.^39,40,57^ The increase in relative abundance of *Peptoniphilus* in PD patients seems to be independent of an entacapone treatment and the inflammation marker calprotectin. However, a significant increase in abundance was observed for patients receiving L-dopa treatment, when compared to the control group, but not for the patients with other dopaminergic treatments. So far, changes in relative abundance of this genus have not been reported before in PD.

*Finegoldia* is also affiliated with the *Clostridiales* family XI. Here, its abundance was found to be relatively increased in PD patients treated with L-dopa, but also in those with elevated calprotectin levels. However, in accordance with previous studies, *Finegoldia* was not significantly different in abundance when the control group was just compared to all PD patients.^14,17,22,30,32^ The genus contains a single described species, *F. magna*, an opportunistic pathogen that can cause infections in immunocompromised hosts.^38^ Members of this genus were also shown to be capable of degrading defensive proteins provided by the host.^58^

Many members of the *Clostridiales* family XI are capable of peptone and amino acid fermentation, including *Peptoniphilus* and *Finegoldia*.^50,51^ It may be speculated that such organisms benefit from an altered protein expression in the colon tissue. For instance, alpha-synuclein has been shown to be pathologically overexpressed in the colon tissue of mice with PD.^59^ In order to valuate this hypothesis, further studies should address the metabolic activity of these genera with respect to distinct proteins and L-dopa, as well as their interactions with the colonic wall and ENS.

Another member of the *Clostridiales* which was decreased relatively in PD, is *Fusicatenibacter*. This genus has been shown to be decreased in organ-specific autoimmune diseases and inflammatory diseases like ulcerative colitis.^42,60^ *Fusicatenibacter* was correlated to fecal secondary bile acids and its numbers decreased under high cholesterol levels.^61^ For secondary bile acids, there is evidence supporting a neuroprotective role in a diverse spectrum of age-related neurodegenerative disorders, including PD.^62^

The family *Bifidobacteriaceae* and the genus *Bifidobacterium* were reported as significantly increased in abundance in PD.^17,22^ In our study, the family *Bifidobacteriaceae* did not show significant differences in the direct comparison of all PD patients to controls. However, its relative abundance was significantly higher in the male PD group and the PD patients treated with entacapone. Bifidobacteria are generally regarded as health promoting, as they have stimulating effects on the immune system and confer resistance to colonization by pathogens, which is the reason why they are widely used for probiotic treatments.^63^

In contrast to Unger and colleagues^17^ and Hopfner and colleagues,^32^ the family *Enterococcaceae* was not found to be different in abundance for the overall comparison of control group and PD. However, this family showed a significant increase in relative abundance in PD patients treated with L-dopa or entacapone. The affiliated genus *Enterococcu*s (especially *Enterococcus faecalis*) was reported to be able to induce irritable bowel syndrome and possesses a variety of immune evasive und protein degradation functionalities.^64–66^ Recently, *Enterococcus faecalis* was also described to convert L-dopa to dopamine in the gut and thus may contribute to its in vivo degradation.^49^

In conclusion, we were able to show that several bacterial genera differed in relative abundance between PD and control samples, in particular under the influence of drug treatments and partly in co-occurrence with the fecal inflammation marker calprotectin.

Within the PD group, we observed a decrease of bacterial taxa presumed as being health-promoting, anti-inflammatory, neuroprotective or having other beneficial effects on the epithelial barrier, such as *Faecalibacterium* and *Fusicatenibacter*. Both genera were decreased in PD patients with elevated calprotectin levels. In addition, we observed an increase in shares of the *Clostridiales* family XI and their affiliated members *Peptoniphilus* and *Finegoldia*, which have been suspected as being opportunistic pathogens in immune compromised hosts. Furthermore, our study confirms the previously reported possible link of COMT inhibitors, like entacapone, with differences in abundance of various microbial taxa. Finally, we provide significant evidence for an influence of L-dopa medication on the relative abundance of several bacterial genera.

Clearly, the causative links between PD, PD medication and the composition and metabolic capacity of the gut microbiota still need to be unraveled in more detail. Gut inflammation, gut motility, and microbial metabolism of PD drugs appear as potential starting points for further investigations, which should be focused more strongly on microbial functionalities than abundances of microbial taxa. Interestingly, prediction of pathways with PICRUSt suggested an increased diversity of biochemical pathways in our PD patients. Especially patients treated with entacapone showed this trend. Such trends appear ideally suited to be validated with, appropriate meta-technologies.^67,68^

Finally, we would like to point out that, besides technical issue (sample size and age, sequencing technology etc.), differences between the findings of our and other studies might also be based on the distinct geographic and cultural background of our study cohort, since the human gut microbiota is regionally different.^69^ Thus, studies worldwide are needed in order to get a more complete picture of potential links between gut microbiota and PD.

## Methods

### Patients and control group

The cohort under investigation comprised 34 PD patients (10 female, 24 male) and 25 healthy controls (14 female, 11 male), who have been investigated previously.^17^ Both groups were age matched. At the time of sampling, the mean age of PD patients was 67.9 (sd = 8.6) years and 63.9 (sd = 5.8) years for the control group, respectively. Special dietary habits or restrictions were not reported. All subjects followed an omnivorous diet. Intakes of antibiotics, probiotics, or prebiotics over the three months prior to the fecal sampling, as well as a history of acute or chronic gastrointestinal disorders were not reported. PD diagnosis was performed according to the UK PD Society Brain Bank Clinical Diagnostic Criteria^70^ by a movement disorder specialist. All PD patients were on dopaminergic drugs (see Supplementary Table 1 for details). Mean duration of the disease was 82 months and the median of the Hoehn and Yahr stage was 2.5.^71^ The control group did not report any pre-existing medical conditions or any chronic or intermittent use of medication.

The study was approved by the ethics committee of the medical association of Saarland and is recorded therein with the identification number 111/12. All enrolled subjects provided written informed consent for their participation.

### Fecal sample collection and DNA isolation

Fecal sampling was performed in 2015. In order to collect samples at home, subjects were provided with sterile containers (MED AUXIL fecal collector set, Süsse, Gudensberg, Germany) and introduced to the collection procedure. Samples were sent to the Institute of Microecology in Herborn, Germany, frozen immediately and then stored at −20 °C until further analysis. Fecal calprotectin concentrations were measured by an enzyme-linked immunosorbent assay as reported previously.^16^

DNA isolation was performed in 2017, using the FastDNA SPIN kit for feces (MP Biomedicals, Heidelberg, Germany), using 150 mg–300 mg of well homogenized fecal. DNA purity and concentration after extraction were measured with an Implen NanoPhotometer P-Class 360 (Implen GmbH, Munich, Germany).

### Library preparation and sequencing

Sequencing library preparation of the V4 and V5 region of the bacterial 16S rRNA genes were performed using the 16S-specific primers 520 F (5′-AYTGGGYDTAAAGNG-3′) and 926 R (5′-CCGTCAATTCMTTTRAGTTT-3′)^72,73^ to produce amplicons of ~380 bp length which is regarded sufficient for identification at genus level.^74^ PCR amplification was performed at least twice per sample. The PCR mixture consisted of 0.5 µl of each primer (10 µM), 0.6 µl of dNTP-Mix (10 mM, each), 5 µl 5× KAPA Hifi Puffer including 20 mM MgCl_2_ (Roche, Mannheim, Germany), 0.1 µl KAPA Hifi Polymerase (Roche), 1 µl DNA template, and was filled up to a final volume of 25 µl with nuclease free water. PCR reactions were performed in a T100 Thermal Cycler (Bio-Rad Laboratories, Munich, Germany) using the following thermal profile: 3 min at 95 °C for initial denaturation, 25 cycles of 30 s at 95 °C for denaturation, 30 s at 55 °C for annealing, and 45 s at 72 °C for elongation, followed by a final elongation step for 5 min at 72 °C. Water-template controls and *Escherichia coli* DNA as positive controls were included for each set of the PCR reaction. Success of PCRs was verified by agarose gel electrophoresis using Midori Green as DNA-dye (Biozym, Olderndorf, Germany). Replicate PCRs of the same sample were pooled and purified with Agencourt AMPure beads (Beckman Coulter, Krefeld, Germany) into 50 µl of 10 mM Tris (pH 8.5) buffer.

Subsequently, a second PCR step was performed to add unique, custom made index barcodes with sequencing adapters to the amplicon targets (detailed in the Supplementary Table 6; Integrated DNA Technologies, Leuven, Belgium) using the same reverse primer as before. The index PCR reaction included 1 µl Ion-index-primer forward and 1 µl 926 R reverse primer with 1.2 µl of dNTP-Mix (10 mM each), 10 µl 5× KAPA Hifi Puffer including 20 mM MgCl_2_ (Roche), 0.2 µl KAPA Hifi Polymerase (Roche), 5 µl amplicon DNA, and was filled up to 50 µl with nuclease free water. PCR reactions were performed in a T100 Thermal Cycler (Bio-Rad Laboratories) using the program detailed above, albeit with 8 cycles. With indices and linker sequences, libraries had a mean sequence length of 428 bp. Purification, quality, and quantity checks, as well as emulsion PCR and following sequencing steps with the Ion PGM Hi-Q OT2 Kit (Thermo Fisher Scientific, Schwerte, Germany) on a Ion PGM sequencer (Thermo Fisher Scientific) were performed following the protocol provided by Kaplan and colleagues and the manufacturer´s instruction.^75^

### Bioinformatics and Statistics

Sequence data were processed using QIIME 1.9.1.^76^ Quality cutoffs were performed using the QIIME standard at Q ≥ 25. Minimum and maximum sequence lengths with the QIIME default of 200 bp and 1000 bp were used. The sequences of four independent runs were merged in a single dataset. Chimeras were removed using vsearch.^77^ Operational taxonomic units (OTU) were chosen within 97% sequence identity. SILVA database release 128 was used to assign taxonomy and align sequences.^78^ Following removal of chloroplast and mitochondrial OTUs, further statistical analyses were made with R version 3.4.3. The phyloseq package version 1.22.3 was used for rarefaction to even sequence depth and exclusion of taxa present in less than 10% of all samples.^79^ Alpha diversity indices for Observed, Chao1, Shannon, and Simpson metrics, as well as beta diversity indices for weighted and unweighted unifrac were also calculated with the phyloseq package. *P*-values were calculated with ANOVA for alpha diversity and ADONIS for beta diversity with the package vegan, version 2.4–6.^80^ The package coin version 1.2–2 was used to compute differences in OTU counts between PD and controls using a two-sided Wilcoxon–Mann–Whitney test for unpaired and non-normally distributed samples in a 10,000 fold Monte-Carlo simulation.^81^ Statistical analyses resulting in *p*-values were corrected using a false discovery rate (FDR) correction for multiple testing.^82^

In order to compare the diversity of metabolic pathways between PD and control samples, a PicRust2^83^ (https://github.com/picrust/picrust2/) analysis was done using the QIIME2^84^ plugin for PicRust2 after export of the necessary files into the QIIME2 format. For the hidden-state prediction (HSP) the method “mp” and for the maximum NSTI the value “2” (optimal for human gut) were chosen. The results file containing the predicted pathway abundances and coverages per sample, based on predicted EC number abundances, was used for calculation of Alpha diversity (Observed, Chao, Shannon and Simpson metrics) and Beta diversity indices and according statistics by the QIIME2^84^ pipeline commands.

Associations of categorical sample attributes were calculated with a *Χ*²-test and validated by Cramér’s V (cV) using the package DescTools version 0.99.27 for R.^85^ Correlations of numeric sample attributes were calculated with a Spearman-*Ρ*.^86^

### Reporting summary

Further information on research design is available in the Nature Research Reporting Summary linked to this article.

## Supplementary information

Supplementary Tables

Reporting Summary Checklist

## Data Availability

Sequences generated and analyzed during this study are accessible at the European Nucleotide Archive (ENA) under the accession number PRJEB30615. Subject data is included in the supplementary information files (Supplementary Table 1). Other datasets are available from the corresponding author on reasonable request.

## Code availability

For this study, no custom functionalities were coded to generate or process the dataset. All used software and packages, their versions, relevant specification and parameters are stated in the “Methods” section in the paragraph “Bioinformatics and Statistics”. Information that is more detailed is available from the corresponding author on reasonable request.

## References

[1] Perez-Pardo P (2017). The gut-brain axis in Parkinson’s disease: possibilities for food-based therapies. Eur. J. Pharmacol..

[2] Fasano A (2013). The role of small intestinal bacterial overgrowth in Parkinson’s disease. Mov. Disord..

[3] Jost WH (2010). Gastrointestinal dysfunction in Parkinson’s disease. J. Neurol. Sci..

[4] Pfeiffer RF (2011). Gastrointestinal dysfunction in Parkinson’s disease. Parkinsonism Relat. Disord..

[5] Savica R (2009). Medical records documentation of constipation preceding Parkinson disease: a case-control study. Neurology.

[6] Chen H (2015). Meta-analyses on prevalence of selected Parkinson’s nonmotor symptoms before and after diagnosis. Transl. Neurodegener..

[7] Abbott RD (2001). Frequency of bowel movements and the future risk of Parkinson’s disease. Neurology.

[8] Gao X, Chen H, Schwarzschild MA, Ascherio A (2011). A prospective study of bowel movement frequency and risk of Parkinson’s disease. Am. J. Epidemiol..

[9] Hawkes CH, Del Tredici K, Braak H (2007). Parkinson’s disease: a dual-hit hypothesis. Neuropathol. Appl. Neurobiol..

[10] Cryan JF, Dinan TG (2012). Mind-altering microorganisms: the impact of the gut microbiota on brain and behaviour. Nat. Rev. Neurosci..

[11] Braak H, Del Tredici K (2017). Neuropathological staging of brain pathology in Sporadic Parkinson’s disease: separating the wheat from the Chaff. J. Parkinsons Dis..

[12] Pan-Montojo F (2010). Progression of Parkinson’s disease pathology is reproduced by intragastric administration of rotenone in mice. PLoS One.

[13] Pan-Montojo F (2012). Environmental toxins trigger PD-like progression via increased alpha-synuclein release from enteric neurons in mice. Sci. Rep..

[14] Keshavarzian A (2015). Colonic bacterial composition in Parkinson’s disease. Mov. Disord..

[15] Forsyth CB (2011). Increased intestinal permeability correlates with sigmoid mucosa alpha-synuclein staining and endotoxin exposure markers in early Parkinson’s disease. PLoS ONE.

[16] Schwiertz Andreas, Spiegel Jörg, Dillmann Ulrich, Grundmann David, Bürmann Jan, Faßbender Klaus, Schäfer Karl-Herbert, Unger Marcus M. (2018). Fecal markers of intestinal inflammation and intestinal permeability are elevated in Parkinson's disease. Parkinsonism & Related Disorders.

[17] Unger MM (2016). Short chain fatty acids and gut microbiota differ between patients with Parkinson’s disease and age-matched controls. Parkinsonism Relat. Disord..

[18] Ge X (2017). Potential role of fecal microbiota from patients with slow transit constipation in the regulation of gastrointestinal motility. Sci. Rep..

[19] Zhu Lixin, Liu Wensheng, Alkhouri Razan, Baker Robert D., Bard Jonathan E., Quigley Eamonn M., Baker Susan S. (2014). Structural changes in the gut microbiome of constipated patients. Physiological Genomics.

[20] van Kessel SP (2019). Gut bacterial tyrosine decarboxylases restrict levels of levodopa in the treatment of Parkinson’s disease. Nat. Commun..

[21] van de Steeg E (2018). An ex vivo fermentation screening platform to study drug metabolism by human gut microbiota. Drug Metab. Disposition.

[22] Hill-Burns EM (2017). Parkinson’s disease and Parkinson’s disease medications have distinct signatures of the gut microbiome. Mov. Disord..

[23] Dorszewska J, Prendecki M, Lianeri M, Kozubski W (2014). Molecular effects of l-dopa therapy in Parkinson’s disease. Curr. Genomics.

[24] Koller WC, Rueda MG (1998). Mechanism of action of dopaminergic agents in Parkinson’s disease. Neurology.

[25] Männistö PT, Kaakkola S (1989). New selective COMT inhibitors: useful adjuncts for Parkinson’s disease?. Trends Pharmacol. Sci..

[26] Sharpless NS, McCann DS (1971). Dopa and 3-O-methyldopa in cerebrospinal fluid of Parkinsonism patients during treatment with oral L-dopa. Clin. Chim. Acta.

[27] Guldberg HC, Marsden CA (1975). Catechol-O-methyl transferase: pharmacological aspects and physiological role. Pharmacol. Rev..

[28] Bonifácio MJ, Palma PN, Almeida L, Soares-da-Silva P (2007). Catechol-O-methyltransferase and its inhibitors in Parkinson’s disease. CNS Drug Rev..

[29] Nissinen E (1988). Inhibition of catechol-O-methyltransferase activity by two novel disubstituted catechols in the rat. Eur. J. Pharmacol..

[30] Scheperjans F (2015). Gut microbiota are related to Parkinson’s disease and clinical phenotype. Mov. Disord..

[31] Li W (2017). Structural changes of gut microbiota in Parkinson’s disease and its correlation with clinical features. Sci. China Life Sci..

[32] Hopfner F (2017). Gut microbiota in Parkinson disease in a northern German cohort. Brain Res..

[33] Bedarf JR (2017). Functional implications of microbial and viral gut metagenome changes in early stage L-DOPA-naïve Parkinson’s disease patients. Genome Med..

[34] Tetz G, Brown SM, Hao Y, Tetz V (2018). Parkinson’s disease and bacteriophages as its overlooked contributors. Sci. Rep..

[35] Smithson M (2006). Confidence Intervals.

[36] Cersosimo MG, Benarroch EE (2012). Pathological correlates of gastrointestinal dysfunction in Parkinson’s disease. Neurobiol. Dis..

[37] Zhao Y, Yu Y-B (2016). Intestinal microbiota and chronic constipation. Springerplus.

[38] Higaki S, Morohashi M (2003). Characteristics of anaerobes from skin specimens. Drugs Exp. Clin. Res..

[39] Jung MY (2014). Peptoniphilus rhinitidis sp. nov., isolated from specimens of chronic rhinosinusitis. Anaerobe.

[40] Cobo F, Rodríguez-Granger J, Sampedro A, Navarro-Marí JM (2017). Peritoneal infection due to Peptoniphilus harei in a patient with intestinal occlusion. Anaerobe.

[41] Rapozo DCM, Bernardazzi C, Souza HSPde (2017). Diet and microbiota in inflammatory bowel disease: The gut in disharmony. World J. Gastroenterol..

[42] Takeshita K (2016). A single species of Clostridium Subcluster XIVa decreased in ulcerative colitis patients. Inflamm. Bowel Dis..

[43] Laval L (2015). Lactobacillus rhamnosus CNCM I-3690 and the commensal bacterium Faecalibacterium prausnitzii A2-165 exhibit similar protective effects to induced barrier hyper-permeability in mice. Gut Microbes.

[44] Martín R (2015). Faecalibacterium prausnitzii prevents physiological damages in a chronic low-grade inflammation murine model. BMC Microbiol..

[45] Maier Eva, Anderson Rachel, Roy Nicole (2017). Live Faecalibacterium prausnitzii Does Not Enhance Epithelial Barrier Integrity in an Apical Anaerobic Co-Culture Model of the Large Intestine. Nutrients.

[46] Becker Anouck, Faßbender Klaus, Oertel Wolfgang H., Unger Marcus M. (2019). A punch in the gut – Intestinal inflammation links environmental factors to neurodegeneration in Parkinson's disease. Parkinsonism & Related Disorders.

[47] Salat D, Tolosa E (2013). Levodopa in the treatment of Parkinson’s disease: current status and new developments. J. Parkinsons Dis..

[48] Goldin BR, Peppercorn MA, Goldman P (1973). Contributions of host and intestinal microflora in the metabolism of L-dopa by the rat. J. Pharmacol. Exp. therapeutics.

[49] Maini Rekdal Vayu, Bess Elizabeth N., Bisanz Jordan E., Turnbaugh Peter J., Balskus Emily P. (2019). Discovery and inhibition of an interspecies gut bacterial pathway for Levodopa metabolism. Science.

[50] Ezaki T (2001). Proposal of the genera Anaerococcus gen. nov., Peptoniphilus gen. nov. and Gallicola gen. nov. for members of the genus Peptostreptococcus. Int. J. Syst. Evol. Microbiol..

[51] Murphy EC, Frick I-M (2013). Gram-positive anaerobic cocci–commensals and opportunistic pathogens. FEMS Microbiol. Rev..

[52] Gordin A, Kaakkola S, Teräväinen H (2004). Clinical advantages of COMT inhibition with entacapone-a review. J Neural Transm.

[53] Kaakkola S (2000). Clinical pharmacology, therapeutic use and potential of COMT inhibitors in Parkinson’s disease. Drugs.

[54] Chen J (2016). Multiple sclerosis patients have a distinct gut microbiota compared to healthy controls. Sci. Rep..

[55] Pisanu A (2018). Neuroinflammation in L-DOPA-induced dyskinesia: beyond the immune function. J Neural Transm.

[56] Wrzosek L (2013). Bacteroides thetaiotaomicron and Faecalibacterium prausnitzii influence the production of mucus glycans and the development of goblet cells in the colonic epithelium of a gnotobiotic model rodent. BMC Biol..

[57] Verma, R., Morrad, S. & Wirtz, J. J. Peptoniphilus asaccharolyticus-associated septic arthritis and osteomyelitis in a woman with osteoarthritis and diabetes mellitus. *BMJ Case Reports* **2017**, 10.1136/bcr-2017-219969 (2017). 10.1136/bcr-2017-219969 PMC5534897 28576913

[58] Frick I-M (2011). Constitutive and inflammation-dependent antimicrobial peptides produced by epithelium are differentially processed and inactivated by the commensal Finegoldia magna and the pathogen Streptococcus pyogenes. J. Immunol..

[59] Kelly LP (2014). Progression of intestinal permeability changes and alpha-synuclein expression in a mouse model of Parkinson’s disease. Mov. Disord..

[60] Zhao F (2018). Alterations of the gut microbiota in Hashimoto’s thyroiditis patients. Thyroid.

[61] Prieto I (2018). Influence of a diet enriched with virgin olive oil or butter on mouse gut microbiota and its correlation to physiological and biochemical parameters related to metabolic syndrome. PLoS ONE.

[62] Ackerman HD, Gerhard GS (2016). Bile acids in neurodegenerative disorders. Front. Aging Neurosci..

[63] Picard C (2005). Review article: bifidobacteria as probiotic agents–physiological effects and clinical benefits. Alimentary Pharmacol. Therapeutics.

[64] Balish E, Warner T (2002). Enterococcus faecalis induces inflammatory bowel disease in interleukin-10 knockout mice. Am. J. Pathol..

[65] Golińska E (2013). Virulence factors of Enterococcus strains isolated from patients with inflammatory bowel disease. World J. Gastroenterol..

[66] Giridhara Upadhyaya PM, Ravikumar KL, Umapathy BL (2009). Review of virulence factors of enterococcus: an emerging nosocomial pathogen. Indian J. Med. Microbiol..

[67] Petriz BA, Franco OL (2017). Metaproteomics as a complementary approach to gut microbiota in health and disease. Front. Chem..

[68] Mondot S, Lepage P (2016). The human gut microbiome and its dysfunctions through the meta-omics prism. Ann. NY Acad. Sci..

[69] He Y (2018). Regional variation limits applications of healthy gut microbiome reference ranges and disease models. Nat. Med..

[70] Massano J, Bhatia KP (2012). Clinical approach to Parkinson’s disease: features, diagnosis, and principles of management. Cold Spring Harb. Perspect. Med..

[71] Hoehn MM, Yahr MD (1967). Parkinsonism: onset, progression and mortality. Neurology.

[72] Claesson MJ (2009). Comparative analysis of pyrosequencing and a phylogenetic microarray for exploring microbial community structures in the human distal intestine. PLoS ONE.

[73] Engelbrektson A (2010). Experimental factors affecting PCR-based estimates of microbial species richness and evenness. ISME J..

[74] Ding L-J, Su J-Q, Xu H-J, Jia Z-J, Zhu Y-G (2015). Long-term nitrogen fertilization of paddy soil shifts iron-reducing microbial community revealed by RNA-(13)C-acetate probing coupled with pyrosequencing. ISME J..

[75] Kaplan H, Ratering S, Felix-Henningsen P, Schnell S (2019). Stability of in situ immobilization of trace metals with different amendments revealed by microbial 13C-labelled wheat root decomposition and efflux-mediated metal resistance of soil bacteria. Sci. Total Environ..

[76] Caporaso JG (2010). QIIME allows analysis of high-throughput community sequencing data. Nat. Methods.

[77] Rognes T, Flouri T, Nichols B, Quince C, Mahé F (2016). VSEARCH: a versatile open source tool for metagenomics. PeerJ.

[78] Pruesse E (2007). SILVA: a comprehensive online resource for quality checked and aligned ribosomal RNA sequence data compatible with ARB. Nucleic Acids Res..

[79] McMurdie PJ, Holmes S (2013). phyloseq: an R package for reproducible interactive analysis and graphics of microbiome census data. PLoS ONE.

[80] Oksanen, J. et al. *vegan: Community Ecology Package. R package version 2.4-6*. https://CRAN.R-project.org/package=vegan (2018).

[81] Hothorn T, Hornik K, van de Wiel, Mark A, Zeileis A (2006). A lego system for conditional inference. Am. Stat..

[82] Benjamini, Y. & Hochberg, Y. Controlling the false discovery rate-a practical and powerful approach to multiple testing. *J. R. Stat. Soc. Ser. A Method*. **57**, 289–300 (1995).

[83] Langille MGI (2013). Predictive functional profiling of microbial communities using 16S rRNA marker gene sequences. Nat. Biotechnol..

[84] Bolyen, E. et al. *QIIME 2: reproducible, interactive, scalable, and extensible microbiome data science.* (2018). 10.1038/s41587-019-0209-9 PMC7015180 31341288

[85] Signorell, Andri et mult. et al. *DescTools: tools for descriptive statistics*. R package version 0.99.27. https://cran.r-project.org/package=DescTools (2019).

[86] Hollander, M., Wolfe, D. A. & Chicken, E. *Nonparametric Statistical Methods* (John Wiley & Sons Inc, Hoboken, New Jersey 2014).

