# Supplementary material for: Effect of Parkinson’s disease and related medications on the composition of the fecal bacterial microbiota
Source: NPJ Parkinsons Dis. 2019 Nov 29;5:28. doi: 10.1038/s41531-019-0100-x (PMC6884491; doi:10.1038/s41531-019-0100-x)
Supplement: Supplementary file 1 — Supplementary Tables [file 41531_2019_100_MOESM1_ESM.pdf]

Supplementary table 1: Summary of individual epidemiological and clinical data

Supplement 1: Summary of individual epidemiological and clinical data of the 34 PD patients and 25 controls, based on supplementary data previously published in Unger et al. (2016)[17], added and changed.

SampleID	Group[1]	Sex	Age	Disease duration (months)	Hoehn-Yahr stage	Phenotype[2]	Calprotectin [µg/g]		Smoker	Appendectomy	Family history for neurodegenerative disorders	Constipation[3]	Entacapone	Gastrointestinal symptoms	Average L-dopa dose[4] (last 2 years)	Other drugs against Parkinson (daily dose)	Drugs affecting gastro-intestinal motility or protone pump inhibitors
IfM P001	PD	f	50	112	3	E	neg.	<20	no	yes	yes	no	no	-	2	14 mg ropinirole	-
IfM P002	PD	m	78	14	2	E	neg.	<20	no	yes	no	no	no	-	1		-
IfM P003	PD	m	65	139	3	HR	neg.	<20	no	no	no	no	yes	-	3	1.05 mg pramipexole<br>4 mg ropinirole	-
IfM P006	PD	m	47	12	2	HR	neg.	<20	no	yes	no	no	no	pyrosis	0	1 mg rasagiline<br>10 mg ropinirole	-
IfM P007	PD	f	74	75	2	E	pos.	109.14	no	no	no	yes	yes	-	2	1 mg rasagiline<br>1.4 mg pramipexole	-
IfM P008	PD	m	60	123	2	T	neg.	<20	no	no	no	no	no	-	0	1 mg rasagiline<br>3.4 mg pramipexole	-
IfM P009	PD	m	68	69	3	T	neg.	<20	no	yes	no	no	no	-	0	1.57 mg pramipexole	-
IfM P010	PD	f	74	108	2	HR	neg.	<20	no	no	yes	yes	yes	pyrosis	1	4 mg ropinirole<br>1 mg rasagiline	40 mg pantoprazole
IfM P011	PD	f	67	168	3	HR	pos.	65.63	no	no	yes	yes	yes	intermittent abdominal pain	2	1 mg rasagiline<br>4.2 mg pramipexole	40 mg pantoprazole
IfM P012	PD	f	78	99	4	E	pos.	345.33	no	no	no	no	no	-	1	1 mg rasagiline	-
IfM P013	PD	m	66	159	4	E	pos.	568.18	no	no	no	no	yes	-	2	50 mg piripedil<br>1 mg rasagiline	-
IfM P014	PD	m	69	147	3	E	neg.	<20	no	no	yes	no	no	-	2	1 mg rasagiline<br>20 mg ropinirole<br>30 mg budipine	-

IfM P015	PD	m	75	159	4	HR	pos.	167.93	no	no	no	no	yes	-	2	1 mg rasagiline<br>2.8 mg pramipexole	-
IfM P016	PD	f	70	39	3	HR	neg.	<20	no	yes	no	no	no	-	1	1.57 mg pramipexole	-
IfM P017	PD	m	72	19	2	E	neg.	<20	no	yes	no	no	no	-	0	1 mg rasagiline	-
IfM P018	PD	m	53	38	2	HR	pos.	371.78	no	no	no	yes	no	-	0	1 mg rasagiline<br>10 mg rotigotine	20 mg domperidone
IfM P019	PD	m	60	84	3	HR	neg.	41.616	no	no	yes	no	yes	-	1	1 mg rasagiline<br>24 mg rotipinole	-
IfM P020	PD	m	69	57	3	E	pos.	97.64	no	no	no	no	yes	pyrosis	1	1 mg rasagiline	20 mg omeprazole
IfM P021	PD	m	78	84	1	HR	neg.	<20	no	yes	yes	no	no	-	0	1 mg rasagiline	-
IfM P022	PD	f	67	144	3	HR	pos.	165.54	no	yes	no	no	no	-	2	1 mg rasagiline<br>3.15 mg pramipexole<br>16 mg ropinorole	15 mg domperidone
IfM P023	PD	m	81	36	3	T	neg.	49.7	no	yes	no	no	no	flatulence	2		-
IfM P024	PD	f	74	72	2	E	pos.	92.3	no	yes	no	no	no	-	1	1 mg rasagiline	-
IfM P025	PD	m	75	228	3	E	neg.	42.09	no	yes	no	no	yes	-	2		-
IfM P026	PD	m	69	15	2	HR	neg.	<20	no	no	no	no	no	-	0	1 mg rasagiline	-
IfM P027	PD	m	53	14	2	E	neg.	<20	no	no	no	no	no	occasional nausea	0	1 mg rasagiline<br>100 mg amantadine	13 g macrogol
IfM P029	PD	f	62	17	3	T	pos.	107.46	yes	yes	no	no	no	pyrosis	0	100 mg pripedil	occasional use of ranitidine
IfM P030	PD	m	61	96	4	HR	neg.	40.902	no	no	yes	yes	no	Pyrosis	3		20 mg pantoprazole
IfM P031	PD	m	74	26	2	E	pos.	146.2	yes	no	yes	no	no	-	1	1 mg rasagiline<br>1.57 mg pramipexole	-
IfM P032	PD	m	59	60	2	HR	neg.	<20	no	yes	no	no	no	-	0	1 mg rasagiline<br>6 mg rotigotine<br>200 mg amantadine	-
IfM P033	PD	m	62	24	2	T	neg.	30.7	no	no	no	yes	no	-	1	1 mg rasagiline<br>200 mg amantadine	-
IfM P034	PD	m	67	60	3	HR	pos.	54	no	yes	no	no	yes	-	2	4 mg rotigotine	20 mg domperidone,<br>26 g macrogol

IfM P035	PD	m	77	48	4	E	neg.	<20	no	no	no	no	no	-	2		-
IfM P036	PD	f	77	87	2	HR	pos.	96.5	no	no	no	yes	yes	-	2	4 mg ropinirole<br>100 mg amantadine	-
IfM P038	PD	f	77	156	3	T	pos.	62.8	no	yes	no	no	no	-	2	0.52 mg pramipexole	-
IfM PK039	Ctrl	f	58	-	-	-	neg.	<20	yes	-	-	no	-	-	-		-
IfM PK040	Ctrl	f	61	-	-	-	neg.	<20	no	-	-	no	-	-	-		-
IfM PK041	Ctrl	f	55	-	-	-	pos.	25.8	no	-	-	no	-	-	-		-
IfM PK042	Ctrl	m	61	-	-	-	neg.	<20	no	-	-	no	-	-	-		-
IfM PK043	Ctrl	f	63	-	-	-	neg.	<20	no	-	-	no	-	-	-		-
IfM PK044	Ctrl	m	66	-	-	-	neg.	<20	yes	-	-	no	-	-	-		-
IfM PK045	Ctrl	f	65	-	-	-	neg.	<20	-	-	-	-	-	-	-		-
IfM PK046	Ctrl	m	64	-	-	-	neg.	<20	yes	-	-	no	-	-	-		-
IfM PK047	Ctrl	m	67	-	-	-	pos.	151	no	-	-	yes	-	-	-		-
IfM PK048	Ctrl	f	63	-	-	-	neg.	<20	yes	-	-	no	-	-	-		-
IfM PK049	Ctrl	m	72	-	-	-	neg.	<20	no	-	-	no	-	-	-		-
IfM PK050	Ctrl	f	70	-	-	-	neg.	<20	no	-	-	no	-	-	-		-
IfM PK051	Ctrl	f	63	-	-	-	neg.	<20	yes	-	-	no	-	-	-		-
IfM PK052	Ctrl	m	59	-	-	-	neg.	<20	yes	-	-	no	-	-	-		-
IfM PK053	Ctrl	f	57	-	-	-	neg.	<20	no	-	-	yes	-	-	-		-
IfM PK054	Ctrl	m	56	-	-	-	neg.	<20	-	-	-	-	-	-	-		-
IfM PK055	Ctrl	f	56	-	-	-	neg.	<20	yes	-	-	no	-	-	-		-
IfM PK056	Ctrl	m	55	-	-	-	neg.	<20	-	-	-	-	-	-	-		-
IfM PK057	Ctrl	m	76	-	-	-	neg.	<20	-	-	-	-	-	-	-		-
IfM PK058	Ctrl	m	71	-	-	-	pos.	27.337	-	-	-	-	-	-	-		-
IfM PK059	Ctrl	f	74	-	-	-	neg.	<20	-	-	-	-	-	-	-		-
IfM PK060	Ctrl	f	66	-	-	-	neg.	<20	no	-	-	no	-	-	-		-
IfM PK061	Ctrl	m	70	-	-	-	neg.	<20	no	-	-	no	-	-	-		-
IfM PK062	Ctrl	f	64	-	-	-	neg.	<20	no	-	-	no	-	-	-		-
IfM PK063	Ctrl	f	64	-	-	-	neg.	<20	-	-	-	-	-	-	-		-

[1] Groups were defined as patients with Parkinson’s disease (PD) and healthy controls (Ctrl).

[2] Phenotype was defined as hypokinetic-rigid (HR), tremor dominant (T) and equivalent (E).

[3] Constipation was defined as less than three bowel movements a week or bowel movements that are hard, dry, small, painful or difficult to pass.

[4] Average daily L-dopa dose in the last two years; 0: 0 mg/day; 1: <300 mg/day; 2: 300-600 mg/day; 3: >600 mg/day.

[17] Unger, M. M. *et al.* Short chain fatty acids and gut microbiota differ between patients with Parkinson's disease and age-matched controls. *Parkinsonism Relat. Disord.* **32**, 66–72; 10.1016/j.parkreldis.2016.08.019 (2016).

Supplementary table 2: PICRUSt analysis of predicted metagenome functionality

		Alpha diversity (Kruskal-Wallis)								Beta diversity	
		Observed		Chao 1		Shannon		Simpson		Bray-Curtis	
		H	p (FDR)	H	p (FDR)	H	p (FDR)	H	p (FDR)	F	p (FDR)
	**Ctrl / PD**	4.08	**0.0435**	3.86	**0.0496**	0.03	0.8540	0.07	0.7943	0.79	0.5730
**Calprotectin**	**Ctrl - / PD -**	1.31	0.3025	1.71	0.3816	0.02	0.9517	0.01	0.9442	0.40	0.9180
	**Ctrl - / PD +**	4.37	0.2195	3.35	0.3816	0.00	0.9517	0.02	0.9442	1.61	0.8040
	**PD - / PD +**	0.47	0.4944	0.54	0.4624	0.00	0.9517	0.00	0.9442	0.85	0.8325
**L-dopa**	**Ctrl / PD -**	0.19	0.6606	0.62	0.5708	1.63	0.3019	1.83	0.2650	1.14	0.3080
	**Ctrl / PD +**	5.72	0.0502	4.54	0.0995	0.21	0.6455	0.16	0.6892	1.17	0.3080
	**PD - / PD +**	0.26	0.6606	0.32	0.5708	2.64	0.3019	2.64	0.2650	1.69	0.3080
**Entacapone**	**Ctrl / PD -**	0.12	0.7254	0.98	0.3218	1.51	0.2195	1.77	0.1831	0.77	0.6000
	**Ctrl / PD +**	15.51	**0.0002**	7.55	**0.0180**	2.66	0.1543	2.66	0.1543	3.33	**0.0030**
	**PD - / PD +**	5.14	**0.0351**	3.19	0.1113	7.52	**0.0183**	6.55	**0.0315**	3.62	**0.0075**
**Phenotype**	**Ctrl / E**	3.54	0.1802	2.00	0.3382	0.32	0.7263	0.23	0.7646	0.98	0.4944
	**Ctrl / HR**	0.20	0.6521	2.32	0.3382	0.27	0.7263	0.12	0.7646	1.04	0.4944
	**Ctrl / T**	4.95	0.1570	1.89	0.3382	6.00	0.0286	5.06	0.0585	2.12	0.1080
	**E / HR**	1.23	0.5359	0.98	0.4830	0.00	0.9816	0.09	0.7646	0.21	0.9930
	**E / T**	0.38	0.6521	0.49	0.5795	6.47	**0.0286**	5.20	0.0585	3.23	0.0780
	**HR / T**	0.26	0.6521	0.15	0.6971	6.60	**0.0286**	4.75	0.0585	2.28	0.1080
**Hoehn- Yahr stage**	**Ctrl /HY 1-2.5**	0.83	0.3616	1.52	0.2183	0.48	0.6689	0.58	0.6680	0.92	0.4970
	**Ctrl / HY 3-4**	6.39	**0.0344**	4.35	0.1113	0.18	0.6689	0.14	0.7083	1.11	0.4970
	**HY 1-2.5 / HY 3-4**	1.42	0.3498	1.54	0.2183	1.07	0.6689	1.14	0.6680	1.26	0.4970

Groups were defined as patients with Parkinson's disease (PD) and healthy controls (Ctrl) and with (+) L-dopa or entacapone medication and calprotectin positivity or without (-).

Phenotype was defined as hypokinetic-rigid (HR), tremor dominant (T) and equivalent (E).

Significant differences ($p < 0.05$) are marked with bold font.

Supplementary table 3: PICRUSt pathway abundance

Groups were compared with a two-sided Wilcoxon-Mann-Whitney test for unpaired and non-normally distributed samples in a 10,000 fold Monte-Carlo simulation.

PD- yes indicates PD patients that were treated with the given medication (L-dopa or entacapone), PD-no indicates PD patients that were not treated with the given medication.

Mean 1 and mean 2 indicate the mean amount of assignments of a pathway in group 1 or group 2, respectively.

SD = standard deviation

M1/M2 = ratio of the means, indicating if the relative abundance of the predicted pathway was higher or lower in a comparison.

p = p-value; p[FDR] = p-value after false discovery rate correction.

Pathway ID = ID of the identified pathways from the MetaCyc database collection (https://biocyc.org/META/organism-summary?object=META).

Pathways with a p[FDR] > 0.05 are not listed. Absence of any pathway with a p[FDR] < 0.05 is indicated by "none".

Group 1	Group 2	mean 1	SD 1	mean 2	SD 2	M1 / M2	p	p [FDR]		Pathway ID
Control	PD	40.75	146.80	176.21	302.77	0.23	0.0001	0.0394	*	VALDEG-PWY

L-dopa

Group 1	Group 2	mean 1	SD 1	mean 2	SD 2	M1 / M2	p	p [FDR]		Pathway ID
Control	PD-yes	none								
Control	PD-no	none								
PD-no	PD-yes	none								

Entacapone

Group 1	Group 2	mean 1	SD 1	mean 2	SD 2	M1 / M2	p	p [FDR]		Pathway ID
Control	PD-yes	none								
Control	PD-no	none								
PD-no	PD-yes	29113.82	21101.77	75088.92	37839.56	0.39	0.0001	0.0393	*	P122-PWY
		42776.89	29946.09	106125.46	50806.51	0.40	0.0002	0.0393	*	P124-PWY
		16609.34	12381.32	57170.93	36652.51	0.29	0.0003	0.0393	*	PWY-6901

Supplementary table 4: PICRUSt EC-abundance

Groups were compared with a two-sided Wilcoxon-Mann-Whitney test for unpaired and non-normally distributed samples in a 10,000 fold Monte-Carlo simulation.

PD- yes indicates PD patients that were treated with the given medication (L-dopa or entacapone), PD-no indicates PD patients that were not treated with the given medication.

Mean 1 and mean 2 indicate the mean amount of assignments of a pathway in group 1 or group 2, respectively.

SD = standard deviation

M1/M2 = ratio of the means, indicating if the relative abundance of the predicted pathway was higher or lower in a comparison.

p = p-value; p[FDR] = p-value after false discovery rate correction.

EC-ID = ID number of the enzyme nomenclature database from ExPASy (https://enzyme.expasy.org/).

Enzymes with a p[FDR] > 0.05 are not listed. Absence of any pathway with a p[FDR] < 0.05 is indicated by "none".

Group 1	Group 2	mean 1	SD 1	mean 2	SD 2	M1 / M2	p	p [FDR]		EC-ID
Control	PD	0.76	1.36	24.53	55.54	0.03	<0.0001	<0.0001	****	EC:2.1.1.104
		85879.35	49309.74	43535.78	26797.98	1.97	<0.0001	<0.0001	****	EC:3.2.1.51
		20879.79	25369.59	7650.01	11525.03	2.73	<0.0001	<0.0001	****	EC:4.2.1.32

L-dopa

Group 1	Group 2	mean 1	SD 1	mean 2	SD 2	M1 / M2	p	p [FDR]		EC-ID
Control	PD-yes	29.29	29.14	624.80	2262.87	0.05	<0.0001	<0.0001	****	EC:1.8.7.1
Control	PD-no	none								
PD-no	PD-yes	none								

Entacapone

Group 1	Group 2	mean 1	SD 1	mean 2	SD 2	M1 / M2	p	p [FDR]		EC-ID
Control	PD-yes	20879.79	25369.59	3208.35	2197.81	6.51	<0.0001	<0.0001	****	EC:4.2.1.32
		24674.16	14527.73	6070.52	4829.35	4.06	<0.0001	<0.0001	****	EC:6.1.1.23
		39.89	36.56	330.20	452.35	0.12	<0.0001	<0.0001	****	EC:1.1.1.67
		5846.14	14884.18	85312.69	155752.81	0.07	<0.0001	<0.0001	****	EC:3.2.1.17
		29.29	29.14	1311.59	3289.08	0.02	<0.0001	<0.0001	****	EC:1.8.7.1
		9800.20	13268.37	908.41	802.94	10.79	0.0001	0.02895	*	EC:2.1.1.242
		48641.97	30582.82	14549.68	16151.41	3.34	0.0001	0.02895	*	EC:1.2.1.2
		7573.23	4973.66	2252.28	2039.72	3.36	0.0002	0.04052	*	EC:1.18.6.1
		44953.09	29149.55	14511.25	18736.28	3.10	0.0002	0.04052	*	EC:4.3.1.15
		2.32	2.12	18.41	22.54	0.13	0.0002	0.04052	*	EC:4.2.2.3
		11757.25	12859.82	2376.15	1917.03	4.95	0.0003	0.04156	*	EC:1.2.1.87
		7.15	11.33	2030.52	6595.01	0.00	0.0003	0.04156	*	EC:1.1.2.8
		9932.89	12539.41	1142.69	1111.84	8.69	0.0004	0.04156	*	EC:1.2.99.7
		34522.74	22718.42	11783.71	12744.69	2.93	0.0004	0.04156	*	EC:3.2.1.177

184843.78	64845.78	105628.05	59292.10	1.75	0.0004	0.04156	*	EC:2.1.1.13
30.72	29.42	178.60	193.54	0.17	0.0004	0.04156	*	EC:2.7.1.175
103.27	304.52	772.68	816.32	0.13	0.0004	0.04156	*	EC:2.5.1.26
33.95	28.91	322.91	532.96	0.11	0.0004	0.04156	*	EC:3.2.1.141
6.37	12.94	245.74	677.19	0.03	0.0004	0.04156	*	EC:6.2.1.34
8808.66	12701.41	724.73	762.58	12.15	0.0005	0.04156	*	EC:2.7.1.14
285.53	230.06	56.61	46.41	5.04	0.0005	0.04156	*	EC:3.2.1.156
16140.94	15010.23	3607.67	2519.03	4.47	0.0005	0.04156	*	EC:2.4.1.4
8734.38	16012.54	24203.01	23972.83	0.36	0.0005	0.04156	*	EC:4.1.3.36
0.15	0.53	11.86	17.70	0.01	0.0005	0.04156	*	EC:2.4.2.30
96897.66	51491.16	45037.13	31704.05	2.15	0.0006	0.04156	*	EC:1.4.7.1
335.11	500.92	856.71	517.85	0.39	0.0006	0.04156	*	EC:2.5.1.32
27267.28	22858.52	73649.05	47181.26	0.37	0.0006	0.04156	*	EC:2.1.1.14
2.81	9.51	20.09	27.85	0.14	0.0006	0.04156	*	EC:2.1.1.86
929.01	2677.81	9829.98	16759.52	0.09	0.0006	0.04156	*	EC:5.1.3.6
10071.64	12866.52	1920.65	2734.63	5.24	0.0007	0.04156	*	EC:4.3.2.3
8669.60	9262.23	40869.56	39700.61	0.21	0.0007	0.04156	*	EC:2.1.1.10
0.32	0.90	2.36	3.98	0.14	0.0007	0.04156	*	EC:1.14.12.1
49777.89	29483.00	23149.48	30581.88	2.15	0.0008	0.04156	*	EC:2.10.1.1
42.01	148.06	42.08	55.57	1.00	0.0008	0.04156	*	EC:4.1.3.17
4275.45	6398.26	16525.92	19793.27	0.26	0.0008	0.04156	*	EC:1.1.1.125
1036.62	3442.92	8674.39	16020.57	0.12	0.0008	0.04156	*	EC:3.1.3.81
749.37	1846.58	9035.89	15929.01	0.08	0.0008	0.04156	*	EC:3.2.1.8
0.76	1.36	33.00	69.55	0.02	0.0008	0.04156	*	EC:2.1.1.104
0.52	1.29	134.36	432.12	0.00	0.0008	0.04156	*	EC:1.1.3.8
13412.95	18226.67	1404.66	1176.71	9.55	0.0009	0.04342	*	EC:1.1.1.271
32519.44	22142.07	11227.68	13322.73	2.90	0.0009	0.04342	*	EC:3.2.1.25
2234.76	4182.33	14394.52	19071.18	0.16	0.0009	0.04342	*	EC:4.2.1.22
8304.94	16012.76	23926.81	24268.80	0.35	0.001	0.04605	*	EC:6.2.1.26
1142.79	1928.96	5414.86	6720.01	0.21	0.001	0.04605	*	EC:3.2.1.58
44174.27	30240.89	18896.96	29617.31	2.34	0.0011	0.0465	*	EC:2.7.7.77
63269.50	44951.50	23449.30	27842.25	2.70	0.0012	0.0465	*	EC:1.17.1.4
18937.84	19350.52	58188.68	42016.82	0.33	0.0012	0.0465	*	EC:2.3.1.12
98.24	314.98	327.76	534.15	0.30	0.0012	0.0465	*	EC:5.4.99.15
1183.31	3466.00	9125.53	15941.21	0.13	0.0012	0.0465	*	EC:2.1.1.35
73294.14	46166.52	26454.01	35890.45	2.77	0.0013	0.0465	*	EC:4.1.1.98
85879.35	49309.74	41241.33	33070.95	2.08	0.0013	0.0465	*	EC:3.2.1.51
643.56	2712.88	563.29	585.68	1.14	0.0013	0.0465	*	EC:3.4.21.26
5720.39	15334.30	9534.98	8047.15	0.60	0.0013	0.0465	*	EC:3.2.1.96
6615.84	18602.32	16737.86	17135.64	0.40	0.0013	0.0465	*	EC:3.1.1.53
1045.50	3441.50	8692.30	16015.52	0.12	0.0013	0.0465	*	EC:3.1.3.4
7.41	19.34	158.89	439.29	0.05	0.0013	0.0465	*	EC:4.2.1.153
5505.33	15214.05	14820.09	18794.20	0.37	0.0014	0.0465	*	EC:3.4.14.5
3019.37	5428.01	13848.75	18224.66	0.22	0.0014	0.0465	*	EC:5.5.1.4
8232.25	13101.97	37907.35	45986.25	0.22	0.0014	0.0465	*	EC:1.6.1.2
1.31	2.72	8.43	16.75	0.16	0.0014	0.0465	*	EC:1.13.11.37
1.13	3.82	19.01	34.81	0.06	0.0014	0.0465	*	EC:5.1.3.25
37216.50	27018.22	13654.59	17403.35	2.73	0.0015	0.04749	*	EC:1.97.1.9
7635.51	15715.79	19337.44	18565.88	0.39	0.0015	0.04749	*	EC:1.1.3.21

		3116.16	5459.92	13420.64	15966.06	0.23	0.0015	0.04749	*	EC:1.1.1.90
		1202.51	2987.42	2879.95	2855.10	0.42	0.0016	0.04768	*	EC:2.7.1.53
		160.47	487.20	743.86	1139.72	0.22	0.0016	0.04768	*	EC:2.7.1.162
		11.07	11.65	206.63	542.44	0.05	0.0016	0.04768	*	EC:1.20.4.3
		1.28	2.67	267.91	862.15	0.00	0.0016	0.04768	*	EC:1.14.13.107
		4577.68	4665.67	294.99	484.16	15.52	0.0017	0.04851	*	EC:3.5.5.1
		39462.79	16717.61	21258.42	13578.60	1.86	0.0017	0.04851	*	EC:2.5.1.105
		2596.97	4777.16	11703.97	15982.47	0.22	0.0017	0.04851	*	EC:3.1.2.20
Control	PD-no	none								
PD-no	PD-yes	17011.09	7946.64	6070.52	4829.35	2.80	<0.0001	<0.0001	****	EC:6.1.1.23
		15099.46	13020.07	51787.03	33861.48	0.29	<0.0001	<0.0001	****	EC:4.1.2.9
		19824.21	17306.45	73649.05	47181.26	0.27	<0.0001	<0.0001	****	EC:2.1.1.14
		5258.14	6983.96	24203.01	23972.83	0.22	<0.0001	<0.0001	****	EC:4.1.3.36
		1051.69	1525.31	6150.64	6517.80	0.17	<0.0001	<0.0001	****	EC:3.2.1.45
		2914.18	2947.42	18279.33	19794.36	0.16	<0.0001	<0.0001	****	EC:5.4.99.29
		2258.56	2894.89	15186.76	17926.24	0.15	<0.0001	<0.0001	****	EC:5.4.99.16
		1062.49	1892.97	8259.13	11693.98	0.13	<0.0001	<0.0001	****	EC:3.1.3.12
		86.90	256.27	772.68	816.32	0.11	<0.0001	<0.0001	****	EC:2.5.1.26
		1050.82	894.61	9357.83	12890.93	0.11	<0.0001	<0.0001	****	EC:2.4.1.57
		1610.28	2149.61	14820.09	18794.20	0.11	<0.0001	<0.0001	****	EC:3.4.14.5
		1304.61	1951.99	13848.75	18224.66	0.09	<0.0001	<0.0001	****	EC:5.5.1.4
		1310.63	2003.28	14394.52	19071.18	0.09	<0.0001	<0.0001	****	EC:4.2.1.22
		16840.57	13438.26	49673.75	26081.14	0.34	0.0001	0.00922	**	EC:2.5.1.74
		15099.46	13020.07	51787.03	33861.48	0.29	0.0001	0.00922	**	EC:4.1.2.22
		2497.89	3449.92	11530.32	12562.85	0.22	0.0001	0.00922	**	EC:2.3.1.241
		4936.92	6953.17	23926.81	24268.80	0.21	0.0001	0.00922	**	EC:6.2.1.26
		49.92	45.37	330.20	452.35	0.15	0.0001	0.00922	**	EC:1.1.1.67
		2131.14	3283.11	16737.86	17135.64	0.13	0.0001	0.00922	**	EC:3.1.1.53
		1419.50	1914.78	13313.38	18456.05	0.11	0.0001	0.00922	**	EC:2.4.1.12
		47.47	57.61	505.29	847.91	0.09	0.0001	0.00922	**	EC:2.4.1.288
		4971.93	12456.69	85312.69	155752.81	0.06	0.0001	0.00922	**	EC:3.2.1.17
		11.38	34.44	36.17	26.16	0.31	0.0002	0.01352	*	EC:4.4.1.25
		15911.62	12906.52	51231.78	41366.68	0.31	0.0002	0.01352	*	EC:3.4.11.2
		2825.23	2531.18	12833.14	9446.87	0.22	0.0002	0.01352	*	EC:5.3.1.5
		616.92	1393.29	2879.95	2855.10	0.21	0.0002	0.01352	*	EC:2.7.1.53
		3979.27	4747.69	27049.79	29780.59	0.15	0.0002	0.01352	*	EC:6.4.1.3
		1065.15	1896.79	8385.69	11629.12	0.13	0.0002	0.01352	*	EC:2.4.1.15
		1.12	1.49	15.17	33.69	0.07	0.0002	0.01352	*	EC:2.7.1.72
		623.74	1566.89	9125.53	15941.21	0.07	0.0002	0.01352	*	EC:2.1.1.35
		2542.88	4015.63	7354.26	6310.56	0.35	0.0003	0.01738	*	EC:4.1.1.15
		1950.52	3163.98	11848.49	16649.38	0.16	0.0003	0.01738	*	EC:3.4.13.22
		827.54	1461.66	5414.86	6720.01	0.15	0.0003	0.01738	*	EC:3.2.1.58
		1903.51	2656.21	14568.57	19189.54	0.13	0.0003	0.01738	*	EC:2.7.7.59
		1830.87	2108.82	15333.63	19946.31	0.12	0.0003	0.01738	*	EC:2.4.99.16
		14705.55	16101.98	58188.68	42016.82	0.25	0.0004	0.01979	*	EC:2.3.1.12
		12408.02	14998.38	50989.00	46810.63	0.24	0.0004	0.01979	*	EC:6.2.1.5

	3357.48	3646.04	16502.12	19801.39	0.20	0.0004	0.01979	*	EC:3.5.4.13
	5186.06	7076.16	37907.35	45986.25	0.14	0.0004	0.01979	*	EC:1.6.1.2
	1680.39	2972.06	13420.64	15966.06	0.13	0.0004	0.01979	*	EC:1.1.1.90
	65.38	144.57	1311.59	3289.08	0.05	0.0004	0.01979	*	EC:1.8.7.1
	163.98	444.53	563.29	585.68	0.29	0.0005	0.02205	*	EC:3.4.21.26
	2914.18	2947.42	18279.33	19794.36	0.16	0.0005	0.02205	*	EC:5.4.99.28
	2334.32	2333.27	16425.43	19347.23	0.14	0.0005	0.02205	*	EC:3.4.21.83
	1852.41	2091.06	15332.98	19941.00	0.12	0.0005	0.02205	*	EC:2.1.1.219
	548.01	1578.99	8674.39	16020.57	0.06	0.0005	0.02205	*	EC:3.1.3.81
	997.72	2990.39	856.71	517.85	1.16	0.0007	0.02784	*	EC:2.5.1.32
	2720.36	2764.41	18164.61	19487.41	0.15	0.0007	0.02784	*	EC:2.7.7.19
	42.78	37.11	327.76	534.15	0.13	0.0007	0.02784	*	EC:5.4.99.15
	1852.41	2091.06	15332.98	19941.00	0.12	0.0007	0.02784	*	EC:2.1.1.220
	550.84	1578.17	8692.30	16015.52	0.06	0.0007	0.02784	*	EC:3.1.3.4
	35.37	33.05	178.60	193.54	0.20	0.0008	0.0295	*	EC:2.7.1.175
	1087.40	1601.22	11703.97	15982.47	0.09	0.0008	0.0295	*	EC:3.1.2.20
	677.57	1656.06	9829.98	16759.52	0.07	0.0008	0.0295	*	EC:5.1.3.6
	0.13	0.46	2.36	3.98	0.06	0.0008	0.0295	*	EC:1.14.12.1
	2974.28	4328.31	17538.20	18478.33	0.17	0.0009	0.03147	*	EC:1.6.5.5
	42.02	34.61	322.91	532.96	0.13	0.0009	0.03147	*	EC:3.2.1.141
	90.34	356.90	743.86	1139.72	0.12	0.0009	0.03147	*	EC:2.7.1.162
	29110.69	23899.24	83046.45	53757.58	0.35	0.001	0.03271	*	EC:6.3.4.18
	16024.28	12133.18	54473.70	42193.47	0.29	0.001	0.03271	*	EC:1.3.98.1
	3753.38	4647.51	13002.35	15505.35	0.29	0.001	0.03271	*	EC:3.1.21.5
	19531.60	14397.17	67882.86	48481.94	0.29	0.001	0.03271	*	EC:3.4.22.40
	4738.53	6292.44	19337.44	18565.88	0.25	0.0011	0.03432	*	EC:1.1.3.21
	3987.91	5692.19	17100.97	18153.95	0.23	0.0011	0.03432	*	EC:5.1.3.32
	145.30	394.01	2860.51	6911.35	0.05	0.0011	0.03432	*	EC:3.4.11.19
	1511.13	3802.41	9314.69	15936.56	0.16	0.0013	0.03995	*	EC:3.1.3.27
	18937.74	16247.50	50978.35	33728.75	0.37	0.0014	0.04176	*	EC:1.6.5.2
	3492.64	4054.61	16520.95	20362.26	0.21	0.0014	0.04176	*	EC:3.1.13.5
	914.82	1315.12	2998.40	3154.86	0.31	0.0016	0.04571	*	EC:5.1.3.15
	2389.44	2963.75	14786.48	17628.96	0.16	0.0016	0.04571	*	EC:2.4.2.6
	2417.90	2686.66	15512.39	20081.72	0.16	0.0016	0.04571	*	EC:2.7.7.42
	665.55	933.89	2274.44	2216.31	0.29	0.0017	0.04789	*	EC:3.2.1.35
	25.39	110.07	27.45	44.63	0.92	0.0018	0.04804	*	EC:2.5.1.46
	381.04	1244.78	876.22	1100.21	0.43	0.0018	0.04804	*	EC:3.4.19.5
	5642.67	7112.29	20323.26	19996.58	0.28	0.0018	0.04804	*	EC:2.2.1.9
	2327.13	2330.09	16286.37	20864.81	0.14	0.0018	0.04804	*	EC:2.7.1.63

Supplementary table 5: PICRUSt KO-abundance

Groups were compared with a two-sided Wilcoxon-Mann-Whitney test for unpaired and non-normally distributed samples in a 10,000 fold Monte-Carlo simulation.

PD- yes indicates PD patients that were treated with the given medication (L-dopa or entacapone), PD-no indicates PD patients that were not treated with the given medication.

Mean 1 and mean 2 indicate the mean amount of assignments of a pathway in group 1 or group 2, respectively.

SD = standard deviation

M1/M2 = ratio of the means, indicating if the relative abundance of the predicted pathway was higher or lower in a comparison.

p = p-value; p[FDR] = p-value after false discovery rate correction.

K0-ID = ID of the enzyme in the KEGG orthology database (https://www.genome.jp/kegg/ko.html).

Enzymes with a p[FDR] > 0.05 are not listed. Absence of any pathway with a p[FDR] < 0.05 is indicated by "none".

Group 1	Group 2	mean 1	SD 1	mean 2	SD 2	M1 / M2	p	p [FDR]		K0-ID
Control	PD	25782.54	18280.69	10911.46	7010.50		<0.0001	<0.0001	****	K04028
		24490.68	14481.18	12273.49	8879.04		<0.0001	<0.0001	****	K07726
		2.29	3.00	53.66	118.10		<0.0001	<0.0001	****	K09700
		9736.48	13234.89	2630.84	4499.12		<0.0001	<0.0001	****	K13889
		63121.45	38258.12	29520.22	25876.77		<0.0001	<0.0001	****	K17836

L-dopa

Group 1	Group 2	mean 1	SD 1	mean 2	SD 2	M1 / M2	p	p [FDR]		K0-ID
Control	PD-yes	2.30	3.87	76.70	183.55		<0.0001	<0.0001	****	K00842
		2.29	3.00	39.68	88.26		<0.0001	<0.0001	****	K09700
		17804.46	14993.90	7199.47	6753.73		<0.0001	<0.0001	****	K19309
Control	PD-no	none								
PD-no	PD-yes	3.90	6.65	8748.76	22769.11		<0.0001	<0.0001	****	K05992

Entacapone

Group 1	Group 2	mean 1	SD 1	mean 2	SD 2	M1 / M2	p	p [FDR]		K0-ID
Control	PD-yes	47111.34	30181.01	12225.33	15637.47	3.85	<0.0001	<0.0001	****	K00123
		171.38	488.03	9491.79	15754.80	0.02	<0.0001	<0.0001	****	K00517
		5845.30	14882.68	85312.69	155752.81	0.07	<0.0001	<0.0001	****	K01185
		44953.09	29149.55	14511.25	18736.28	3.10	<0.0001	<0.0001	****	K01751
		11350.54	8652.29	36838.11	20610.76	0.31	<0.0001	<0.0001	****	K02068
		25782.54	18280.69	7998.31	6649.09	3.22	<0.0001	<0.0001	****	K04028
		10.97	9.38	54.27	45.42	0.20	<0.0001	<0.0001	****	K05299
		9931.81	12538.19	1130.89	1116.23	8.78	<0.0001	<0.0001	****	K05303
		372.99	970.13	17266.95	32132.81	0.02	<0.0001	<0.0001	****	K05992
		158.51	506.53	8659.59	15991.86	0.02	<0.0001	<0.0001	****	K07016

4628.43	14350.61	20120.52	27736.17	0.23	<0.0001	<0.0001	****	K07341
9932.89	12539.41	1142.69	1111.84	8.69	<0.0001	<0.0001	****	K07469
137.01	462.51	8395.09	15412.09	0.02	<0.0001	<0.0001	****	K07487
185.64	506.34	8694.55	15509.42	0.02	<0.0001	<0.0001	****	K07505
24490.68	14481.18	6127.23	3223.71	4.00	<0.0001	<0.0001	****	K07726
5708.02	6855.63	32568.35	30835.04	0.18	<0.0001	<0.0001	****	K07778
2098.16	6405.85	9211.81	15794.43	0.23	<0.0001	<0.0001	****	K08368
5959.72	7650.90	39046.01	36016.65	0.15	<0.0001	<0.0001	****	K08969
0.23	0.91	12.05	17.72	0.02	<0.0001	<0.0001	****	K09132
24674.16	14527.73	6070.52	4829.35	4.06	<0.0001	<0.0001	****	K09759
1997.27	2668.85	19204.54	22804.37	0.10	<0.0001	<0.0001	****	K09952
441.85	744.34	9617.87	16531.74	0.05	<0.0001	<0.0001	****	K12990
9736.48	13234.89	889.46	878.24	10.95	<0.0001	<0.0001	****	K13889
9767.22	13249.45	942.43	856.90	10.36	<0.0001	<0.0001	****	K13890
2.88	9.49	20.73	27.40	0.14	<0.0001	<0.0001	****	K14084
9800.20	13268.37	908.41	802.94	10.79	<0.0001	<0.0001	****	K15984
3415.77	4329.62	28211.43	32253.49	0.12	<0.0001	<0.0001	****	K16235
243.10	491.72	9030.70	16040.82	0.03	<0.0001	<0.0001	****	K18934
158.51	506.53	8659.59	15991.86	0.02	<0.0001	<0.0001	****	K19138
158.51	506.53	8659.59	15991.86	0.02	<0.0001	<0.0001	****	K19139
281.15	962.05	17034.23	31408.57	0.02	<0.0001	<0.0001	****	K19140
17804.46	14993.90	4029.18	1900.46	4.42	<0.0001	<0.0001	****	K19309
26656.40	27067.52	4696.40	2483.74	5.68	<0.0001	<0.0001	****	K19310
39.89	36.56	330.20	452.35	0.12	0.0001	0.0131	*	K00045
29.29	29.14	1311.59	3289.08	0.02	0.0001	0.0131	*	K00392
103.27	304.52	772.68	816.32	0.13	0.0001	0.0131	*	K00803
10153.25	10178.65	40974.63	37239.16	0.25	0.0001	0.0131	*	K00818
2.30	3.87	151.12	256.76	0.02	0.0001	0.0131	*	K00842
33.95	28.91	322.91	532.96	0.11	0.0001	0.0131	*	K01236
25.62	24.58	127.03	152.07	0.20	0.0001	0.0131	*	K01820
1005.46	4057.81	17084.51	31447.80	0.06	0.0001	0.0131	*	K02840
42879.23	23202.33	15302.04	18849.50	2.80	0.0001	0.0131	*	K03186
157885.83	70645.92	64653.65	50942.38	2.44	0.0001	0.0131	*	K03292
9933.80	12539.34	1149.88	1107.20	8.64	0.0001	0.0131	*	K06937
128174.18	59748.63	61661.36	41849.72	2.08	0.0001	0.0131	*	K07003
18951.24	17901.17	56151.15	33447.62	0.34	0.0001	0.0131	*	K07586
9658.03	13246.51	766.69	842.91	12.60	0.0001	0.0131	*	K07709
9760.54	13242.96	926.74	871.03	10.53	0.0001	0.0131	*	K13891
564.16	2656.12	283.40	313.66	1.99	0.0001	0.0131	*	K15876
30.72	29.42	178.60	193.54	0.17	0.0001	0.0131	*	K16146
96897.66	51491.16	45037.13	31704.05	2.15	0.0002	0.0222	*	K00284
2234.76	4182.33	14394.52	19071.18	0.16	0.0002	0.0222	*	K01697
11747.58	12667.04	2274.17	1664.52	5.17	0.0002	0.0222	*	K04024
254.60	522.77	2022.41	3266.82	0.13	0.0002	0.0222	*	K06990
35301.46	24312.74	10916.69	13821.91	3.23	0.0002	0.0222	*	K07219
5444.46	15279.71	16823.06	18507.89	0.32	0.0002	0.0222	*	K07498
0.15	0.53	11.86	17.70	0.01	0.0002	0.0222	*	K10798
8808.66	12701.41	724.73	762.58	12.15	0.0002	0.0222	*	K11214
10392.01	16228.54	36170.84	33253.09	0.29	0.0002	0.0222	*	K18891

989.34	3157.61	14123.43	21743.77	0.07	0.0003	0.0269	*	K00299
27267.28	22858.52	73649.05	47181.26	0.37	0.0003	0.0269	*	K00549
13412.95	18226.67	1404.66	1176.71	9.55	0.0003	0.0269	*	K02377
1493.36	2943.61	9732.28	15741.19	0.15	0.0003	0.0269	*	K03319
10311.55	12727.32	1455.67	1094.28	7.08	0.0003	0.0269	*	K03779
4154.67	10913.95	15000.61	17888.95	0.28	0.0003	0.0269	*	K03827
29981.82	26871.41	7203.41	6381.60	4.16	0.0003	0.0269	*	K04031
577.91	992.38	17083.55	30701.48	0.03	0.0003	0.0269	*	K06610
307334.27	145167.37	156729.05	101417.35	1.96	0.0003	0.0269	*	K07718
2229.14	4197.81	14544.76	19239.54	0.15	0.0003	0.0269	*	K07768
4305.61	8010.47	27927.55	37431.46	0.15	0.0003	0.0269	*	K10005
285.53	230.06	56.61	46.41	5.04	0.0003	0.0269	*	K15531
1599.56	3820.97	8003.74	11678.30	0.20	0.0003	0.0269	*	K16055
317.83	580.89	9199.55	15999.64	0.03	0.0003	0.0269	*	K19165
184843.78	64845.78	105628.05	59292.10	1.75	0.0004	0.0301	*	K00548
2.81	9.51	20.09	27.85	0.14	0.0004	0.0301	*	K00584
643.56	2712.88	563.29	585.68	1.14	0.0004	0.0301	*	K01322
2.32	2.12	18.41	22.54	0.13	0.0004	0.0301	*	K01729
16140.94	15010.23	3607.67	2519.03	4.47	0.0004	0.0301	*	K05341
8787.30	12716.89	743.68	766.66	11.82	0.0004	0.0301	*	K08084
42.01	148.06	42.08	55.57	1.00	0.0004	0.0301	*	K10218
28.35	27.86	167.09	178.78	0.17	0.0004	0.0301	*	K10232
390.75	1727.11	335.25	452.11	1.17	0.0004	0.0301	*	K13668
32.84	31.02	417.81	854.03	0.08	0.0004	0.0301	*	K13671
20539.14	13950.21	6947.02	5812.79	2.96	0.0004	0.0301	*	K13954
29941.24	22543.63	9163.01	13064.81	3.27	0.0004	0.0301	*	K16248
9471.29	12707.00	753.49	881.58	12.57	0.0004	0.0301	*	K18843
7.05	10.25	606.39	1817.43	0.01	0.0004	0.0301	*	K19545
7.15	11.33	2030.52	6595.01	0.00	0.0005	0.0334	*	K00114
8669.60	9262.23	40869.56	39700.61	0.21	0.0005	0.0334	*	K00547
30000.05	22591.17	9116.81	13009.71	3.29	0.0005	0.0334	*	K02099
7251.20	4975.66	1958.78	1643.28	3.70	0.0005	0.0334	*	K02588
15483.94	17342.05	51099.58	38153.82	0.30	0.0005	0.0334	*	K04047
68148.53	32332.74	33351.05	17876.22	2.04	0.0005	0.0334	*	K08978
2.29	3.00	61.99	126.98	0.04	0.0005	0.0334	*	K09700
2291.82	4212.41	14552.30	19215.54	0.16	0.0005	0.0334	*	K10006
2291.86	4212.46	14552.51	19215.57	0.16	0.0005	0.0334	*	K10007
2293.18	4212.35	16551.33	19344.88	0.14	0.0005	0.0334	*	K10008
5.14	6.70	30.13	61.74	0.17	0.0005	0.0334	*	K13485
1046.44	3449.20	8860.18	15986.63	0.12	0.0006	0.0361	*	K00557
335.11	500.92	856.71	517.85	0.39	0.0006	0.0361	*	K02291
17944.53	18053.50	55716.79	33335.33	0.32	0.0006	0.0361	*	K07570
73246.07	38404.72	34760.41	18950.15	2.11	0.0006	0.0361	*	K07814
45.74	149.39	110.53	166.64	0.41	0.0006	0.0361	*	K11312
11756.41	12859.39	2372.79	1919.49	4.95	0.0006	0.0361	*	K13922
8812.04	12735.75	822.16	753.31	10.72	0.0006	0.0361	*	K14048
102.91	187.53	476.72	425.65	0.22	0.0006	0.0361	*	K16906
1.13	3.82	19.01	34.81	0.06	0.0006	0.0361	*	K17947
16824.13	16687.25	4565.13	2262.95	3.69	0.0006	0.0361	*	K18369

0.31	1.21	0.89	0.82	0.35	0.0006	0.0361	*	K19273
8734.38	16012.54	24203.01	23972.83	0.36	0.0007	0.0382	*	K01661
198860.79	76133.76	115361.84	56894.56	1.72	0.0007	0.0382	*	K02315
49777.89	29483.00	23149.48	30581.88	2.15	0.0007	0.0382	*	K03750
95195.85	72509.82	31212.14	54370.51	3.05	0.0007	0.0382	*	K05795
309005.70	151689.59	155394.61	105580.78	1.99	0.0007	0.0382	*	K07720
929.01	2677.81	9829.98	16759.52	0.09	0.0007	0.0382	*	K08679
21.72	20.03	193.68	321.94	0.11	0.0007	0.0382	*	K11708
6.37	12.94	245.74	677.19	0.03	0.0007	0.0382	*	K12508
10001.96	12808.32	1291.99	1189.61	7.74	0.0007	0.0382	*	K16139
8309.15	11607.06	24525.00	16594.12	0.34	0.0007	0.0382	*	K16209
1479.93	2325.67	6578.01	7297.14	0.22	0.0007	0.0382	*	K18907
4275.45	6398.26	16525.92	19793.27	0.26	0.0008	0.0400	*	K00065
749.37	1846.58	9035.89	15929.01	0.08	0.0008	0.0400	*	K01181
1142.79	1928.96	5414.86	6720.01	0.21	0.0008	0.0400	*	K01210
28978.32	22852.21	8887.10	13418.64	3.26	0.0008	0.0400	*	K01430
34522.74	22718.42	11783.71	12744.69	2.93	0.0008	0.0400	*	K01811
8304.94	16012.76	23926.81	24268.80	0.35	0.0008	0.0400	*	K01911
197.74	493.30	8400.73	15406.14	0.02	0.0008	0.0400	*	K02538
10350.29	12730.66	1563.76	1116.17	6.62	0.0008	0.0400	*	K03780
9888.95	13234.71	3054.48	7411.82	3.24	0.0008	0.0400	*	K04088
2662.13	4917.60	11549.68	15335.16	0.23	0.0008	0.0400	*	K12293
77.08	210.29	125.18	144.04	0.62	0.0008	0.0400	*	K17640
3458.50	4492.29	21117.16	23271.10	0.16	0.0009	0.0415	*	K00355
1202.51	2987.42	2879.95	2855.10	0.42	0.0009	0.0415	*	K00880
1036.62	3442.92	8674.39	16020.57	0.12	0.0009	0.0415	*	K01096
32519.44	22142.07	11227.68	13322.73	2.90	0.0009	0.0415	*	K01192
1057.11	1899.44	5368.45	6703.22	0.20	0.0009	0.0415	*	K02671
28950.16	22889.05	8609.62	12733.12	3.36	0.0009	0.0415	*	K03191
28.69	27.10	261.14	424.48	0.11	0.0009	0.0415	*	K04756
98.24	314.98	327.76	534.15	0.30	0.0009	0.0415	*	K06044
6.77	23.82	31.20	35.59	0.22	0.0009	0.0415	*	K07445
161.33	299.22	603.26	521.94	0.27	0.0009	0.0415	*	K09703
21.60	19.99	193.68	322.19	0.11	0.0009	0.0415	*	K11709
3116.16	5459.92	13420.64	15966.06	0.23	0.0010	0.0437	*	K00055
29051.76	22887.14	9050.41	13336.93	3.21	0.0010	0.0437	*	K01428
9663.94	10134.81	35390.35	27072.87	0.27	0.0010	0.0437	*	K03449
75805.40	42424.87	31693.60	18112.55	2.39	0.0010	0.0437	*	K06133
1315.30	2471.73	6495.20	7832.42	0.20	0.0010	0.0437	*	K16784
63121.45	38258.12	25937.00	30720.30	2.43	0.0010	0.0437	*	K17836
29235.50	22772.84	9425.72	12632.64	3.10	0.0010	0.0437	*	K18345
35355.02	47876.77	7614.69	10860.68	4.64	0.0010	0.0437	*	K19091
0.52	1.29	134.36	432.12	0.00	0.0011	0.0448	*	K00103
0.76	1.36	33.00	69.55	0.02	0.0011	0.0448	*	K00588
642.93	784.78	9187.53	16147.91	0.07	0.0011	0.0448	*	K00924
29019.20	22887.55	8979.34	13324.35	3.23	0.0011	0.0448	*	K03188
979.17	1893.18	2738.99	2512.18	0.36	0.0011	0.0448	*	K03929
325.49	628.15	9035.11	16203.66	0.04	0.0011	0.0448	*	K06909
4339.19	5643.79	18944.09	21128.06	0.23	0.0011	0.0448	*	K07693

		12721.92	17875.64	38774.94	32037.79	0.33	0.0011	0.0448	*	K08987
		55.91	156.97	322.20	424.67	0.17	0.0011	0.0448	*	K09740
		30039.75	22338.13	9887.39	12972.73	3.04	0.0011	0.0448	*	K13479
		28831.03	22857.75	8536.56	12769.40	3.38	0.0011	0.0448	*	K17236
		29019.20	22887.55	8972.75	13305.72	3.23	0.0012	0.0485	*	K03189
Control	PD-no	none								
PD-no	PD-yes	303.20	1087.71	9491.79	15754.80	0.03	<0.0001	<0.0001	****	K00517
		19824.21	17306.45	73649.05	47181.26	0.27	<0.0001	<0.0001	****	K00549
		1051.69	1525.31	6150.54	6517.61	0.17	<0.0001	<0.0001	****	K01201
		1610.28	2149.61	14820.09	18794.20	0.11	<0.0001	<0.0001	****	K01278
		15099.46	13020.07	51787.03	33861.48	0.29	<0.0001	<0.0001	****	K01621
		1310.63	2003.28	14394.52	19071.18	0.09	<0.0001	<0.0001	****	K01697
		27.90	27.39	127.03	152.07	0.22	<0.0001	<0.0001	****	K01820
		1304.61	1951.99	13848.75	18224.66	0.09	<0.0001	<0.0001	****	K01858
		9456.00	8019.83	36838.11	20610.76	0.26	<0.0001	<0.0001	****	K02068
		973.36	2664.03	17084.51	31447.80	0.06	<0.0001	<0.0001	****	K02840
		5950.27	7429.42	35390.35	27072.87	0.17	<0.0001	<0.0001	****	K03449
		1869.53	3278.72	15000.61	17888.95	0.12	<0.0001	<0.0001	****	K03827
		2131.14	3283.11	16737.86	17135.64	0.13	<0.0001	<0.0001	****	K05970
		3221.67	4032.76	21361.38	20352.13	0.15	<0.0001	<0.0001	****	K07006
		10572.31	11268.56	46634.61	33854.92	0.23	<0.0001	<0.0001	****	K07118
		3091.73	5125.18	16823.06	18507.89	0.18	<0.0001	<0.0001	****	K07498
		290.09	1046.30	8694.55	15509.42	0.03	<0.0001	<0.0001	****	K07505
		15322.58	12419.77	55716.79	33335.33	0.28	<0.0001	<0.0001	****	K07570
		1659.79	2030.70	14981.71	19850.18	0.11	<0.0001	<0.0001	****	K07776
		5404.28	7290.25	39046.01	36016.65	0.14	<0.0001	<0.0001	****	K08969
		252.14	870.11	603.26	521.94	0.42	<0.0001	<0.0001	****	K09703
		1410.78	2013.39	16551.33	19344.88	0.09	<0.0001	<0.0001	****	K10008
		516.20	1102.76	9617.87	16531.74	0.05	<0.0001	<0.0001	****	K12990
		4481.33	6636.53	24525.00	16594.12	0.18	<0.0001	<0.0001	****	K16209
		3697.19	4594.33	28211.43	32253.49	0.13	<0.0001	<0.0001	****	K16235
		47.47	57.61	505.29	847.91	0.09	<0.0001	<0.0001	****	K16650
		10.96	21.70	125.18	144.04	0.09	<0.0001	<0.0001	****	K17640
		11.38	34.44	36.17	26.16	0.31	<0.0001	<0.0001	****	K17950
		5984.00	6509.24	36170.84	33253.09	0.17	<0.0001	<0.0001	****	K18891
		5711.09	6334.38	27796.20	21215.26	0.21	<0.0001	<0.0001	****	K18892
		3080.22	3424.92	13687.15	15631.96	0.23	<0.0001	<0.0001	****	K18929
		374.75	1122.44	9030.70	16040.82	0.04	<0.0001	<0.0001	****	K18934
		291.58	1084.44	8659.59	15991.86	0.03	<0.0001	<0.0001	****	K19138
		65.38	144.57	1311.59	3289.08	0.05	0.0001	0.0098	**	K00392
		3099.49	3431.37	13760.49	15652.55	0.23	0.0001	0.0098	**	K00782
		86.90	256.27	772.68	816.32	0.11	0.0001	0.0098	**	K00803
		616.92	1393.29	2879.95	2855.10	0.21	0.0001	0.0098	**	K00880
		2720.36	2764.41	18164.61	19487.41	0.15	0.0001	0.0098	**	K00970
		4971.75	12456.25	85312.69	155752.81	0.06	0.0001	0.0098	**	K01185
		3670.62	4285.12	30439.38	38910.49	0.12	0.0001	0.0098	**	K01802

4936.92	6953.17	23926.81	24268.80	0.21	0.0001	0.0098	**	K01911
2497.89	3449.92	11530.32	12562.85	0.22	0.0001	0.0098	**	K02517
319.33	1057.42	8400.73	15406.14	0.04	0.0001	0.0098	**	K02538
709.50	1463.78	5368.45	6703.22	0.13	0.0001	0.0098	**	K02671
1454.34	4008.89	9732.28	15741.19	0.15	0.0001	0.0098	**	K03319
3608.02	3850.46	18017.40	19854.23	0.20	0.0001	0.0098	**	K03587
12060.02	13712.48	51099.58	38153.82	0.24	0.0001	0.0098	**	K04047
1.12	1.49	15.17	33.69	0.07	0.0001	0.0098	**	K04343
2914.18	2947.42	18279.33	19794.36	0.16	0.0001	0.0098	**	K06177
136.64	296.26	1258.97	1498.33	0.11	0.0001	0.0098	**	K07002
291.58	1084.44	8659.59	15991.86	0.03	0.0001	0.0098	**	K07016
3031.13	2969.95	18944.09	21128.06	0.16	0.0001	0.0098	**	K07693
2473.48	2674.65	16584.29	19721.81	0.15	0.0001	0.0098	**	K08296
1729.62	3027.64	11510.06	16626.09	0.15	0.0001	0.0098	**	K08641
8910.91	9809.35	38774.94	32037.79	0.23	0.0001	0.0098	**	K08987
2432.67	2013.34	14091.48	15813.43	0.17	0.0001	0.0098	**	K08996
1389.22	2001.10	14552.51	19215.57	0.10	0.0001	0.0098	**	K10007
1662.38	2308.48	15338.88	19307.67	0.11	0.0001	0.0098	**	K10805
1823.63	2108.73	15277.77	19935.29	0.12	0.0001	0.0098	**	K11533
2081.18	2799.03	20501.34	29746.83	0.10	0.0001	0.0098	**	K12292
2200.82	2100.63	16757.54	20009.69	0.13	0.0001	0.0098	**	K13787
525.99	660.46	8003.74	11678.30	0.07	0.0001	0.0098	**	K16055
16.43	28.15	346.85	633.97	0.05	0.0001	0.0098	**	K16238
11941.29	9863.83	36506.08	22138.58	0.33	0.0001	0.0098	**	K18926
3079.77	3423.32	13690.88	15630.21	0.22	0.0001	0.0098	**	K18928
291.58	1084.44	8659.59	15991.86	0.03	0.0001	0.0098	**	K19139
564.47	2134.03	17034.23	31408.57	0.03	0.0001	0.0098	**	K19140
49.92	45.37	330.20	452.35	0.15	0.0002	0.0152	*	K00045
2974.28	4328.31	17538.20	18478.33	0.17	0.0002	0.0152	*	K00344
8732.37	8146.00	40974.63	37239.16	0.21	0.0002	0.0152	*	K00818
5258.14	6983.96	24203.01	23972.83	0.22	0.0002	0.0152	*	K01661
14546.17	11486.10	53520.51	39595.20	0.27	0.0002	0.0152	*	K02077
31734.72	26366.71	96159.41	58169.81	0.33	0.0002	0.0152	*	K03293
2154.09	2367.37	15744.26	19988.99	0.14	0.0002	0.0152	*	K03578
872.73	2510.08	17266.95	32132.81	0.05	0.0002	0.0152	*	K05992
15213.01	9252.59	6127.23	3223.71	2.48	0.0002	0.0152	*	K07726
1352.00	2005.21	14544.76	19239.54	0.09	0.0002	0.0152	*	K07768
5297.88	6497.25	32568.35	30835.04	0.16	0.0002	0.0152	*	K07778
1726.58	2283.62	14530.69	18964.06	0.12	0.0002	0.0152	*	K08156
1288.76	2674.12	9211.81	15794.43	0.14	0.0002	0.0152	*	K08368
2597.48	3949.01	27927.55	37431.46	0.09	0.0002	0.0152	*	K10005
356.71	1225.35	659.40	842.03	0.54	0.0002	0.0152	*	K10545
2537.56	3479.10	20818.90	26471.24	0.12	0.0002	0.0152	*	K11263
2720.07	3110.56	14918.44	15478.39	0.18	0.0002	0.0152	*	K13256
826.57	1485.89	6495.20	7832.42	0.13	0.0002	0.0152	*	K16784
1.65	2.58	41.59	60.83	0.04	0.0002	0.0152	*	K18333
1680.39	2972.06	13420.64	15966.06	0.13	0.0003	0.0190	*	K00055
1357.72	4432.07	9187.53	16147.91	0.15	0.0003	0.0190	*	K00924
1087.40	1601.22	11703.97	15982.47	0.09	0.0003	0.0190	*	K01073

548.01	1578.99	8674.39	16020.57	0.06	0.0003	0.0190	*	K01096
2334.32	2333.27	16425.43	19347.23	0.14	0.0003	0.0190	*	K01354
16840.57	13438.26	49673.75	26081.14	0.34	0.0003	0.0190	*	K02548
17838.48	13738.25	53351.50	38970.81	0.33	0.0003	0.0190	*	K03799
18772.57	14998.48	63396.00	41680.32	0.30	0.0003	0.0190	*	K07177
4641.36	7254.98	14215.22	15894.09	0.33	0.0003	0.0190	*	K07316
1852.41	2091.06	15332.98	19941.00	0.12	0.0003	0.0190	*	K07442
15320.70	12320.98	56151.15	33447.62	0.27	0.0003	0.0190	*	K07586
17011.09	7946.64	6070.52	4829.35	2.80	0.0003	0.0190	*	K09759
2829.72	4401.08	19204.54	22804.37	0.15	0.0003	0.0190	*	K09952
1389.18	2000.93	14552.30	19215.54	0.10	0.0003	0.0190	*	K10006
7.57	29.38	20.73	27.40	0.36	0.0003	0.0190	*	K14084
1175.84	1957.73	6578.01	7297.14	0.18	0.0003	0.0190	*	K18907
25.88	36.48	147.65	144.79	0.18	0.0003	0.0190	*	K19426
4738.53	6292.44	19337.44	18565.88	0.25	0.0004	0.0214	*	K00105
1997.62	2654.77	15387.72	19264.78	0.13	0.0004	0.0214	*	K00325
594.15	1575.19	8860.18	15986.63	0.07	0.0004	0.0214	*	K00557
14705.55	16101.98	58188.68	42016.82	0.25	0.0004	0.0214	*	K00627
2170.05	2467.02	15562.20	19792.54	0.14	0.0004	0.0214	*	K01057
120.24	355.14	906.63	1180.99	0.13	0.0004	0.0214	*	K01758
6204.03	7499.20	25494.18	23404.93	0.24	0.0004	0.0214	*	K01902
2.79	9.92	7.59	7.40	0.37	0.0004	0.0214	*	K02812
2258.56	2894.89	15186.76	17926.24	0.15	0.0004	0.0214	*	K05343
322.96	718.12	2022.41	3266.82	0.16	0.0004	0.0214	*	K06990
1841.88	2083.26	15006.29	19904.52	0.12	0.0004	0.0214	*	K07230
2289.42	2345.46	19811.78	22307.26	0.12	0.0004	0.0214	*	K07259
32.07	30.45	167.09	178.78	0.19	0.0004	0.0214	*	K10232
1848.40	2112.97	15437.58	20073.17	0.12	0.0004	0.0214	*	K13527
3696.80	4225.93	30875.16	40146.34	0.12	0.0004	0.0214	*	K13571
65.18	108.34	283.40	313.66	0.23	0.0004	0.0214	*	K15876
1830.87	2108.82	15333.63	19946.31	0.12	0.0004	0.0214	*	K16147
444.54	1071.02	9199.55	15999.64	0.05	0.0004	0.0214	*	K19165
8.91	21.60	20.57	17.47	0.43	0.0004	0.0214	*	K19166
1419.50	1914.78	13313.38	18456.05	0.11	0.0005	0.0251	*	K00694
827.54	1461.66	5414.86	6720.01	0.15	0.0005	0.0251	*	K01210
3357.48	3646.04	16502.12	19801.39	0.20	0.0005	0.0251	*	K01494
2542.88	4015.63	7354.26	6310.56	0.35	0.0005	0.0251	*	K01580
997.72	2990.39	856.71	517.85	1.16	0.0005	0.0251	*	K02291
277.78	1048.86	8395.09	15412.09	0.03	0.0005	0.0251	*	K07487
1852.35	2090.91	15330.77	19943.63	0.12	0.0005	0.0251	*	K09009
1848.40	2112.97	15437.58	20073.17	0.12	0.0005	0.0251	*	K13570
163.98	444.53	563.29	585.68	0.29	0.0006	0.0284	*	K01322
19531.60	14397.17	67882.86	48481.94	0.29	0.0006	0.0284	*	K01372
16614.11	13329.97	47944.99	25504.89	0.35	0.0006	0.0284	*	K01760
11477.48	10148.48	35355.60	24542.59	0.32	0.0006	0.0284	*	K02074
1828.60	2108.27	15329.82	19943.82	0.12	0.0006	0.0284	*	K07503
1163.29	1561.91	11549.68	15335.16	0.10	0.0006	0.0284	*	K12293
113.78	516.18	86.24	84.49	1.32	0.0006	0.0284	*	K17245
40.83	128.00	190.15	363.19	0.21	0.0006	0.0284	*	K18930

3188.43	4460.92	22519.64	26762.90	0.14	0.0007	0.0307	*	K00324
2825.23	2531.18	12833.14	9446.87	0.22	0.0007	0.0307	*	K01805
11478.17	10147.48	35355.20	24544.37	0.32	0.0007	0.0307	*	K02075
3987.91	5692.19	17100.97	18153.95	0.23	0.0007	0.0307	*	K03534
15995.94	12663.48	43140.45	28844.46	0.37	0.0007	0.0307	*	K03975
18113.21	14700.97	58906.69	43228.43	0.31	0.0007	0.0307	*	K06191
780.15	2137.81	17083.55	30701.48	0.05	0.0007	0.0307	*	K06610
7638.29	9585.86	42380.70	40501.07	0.18	0.0007	0.0307	*	K06999
4.45	3.73	48.46	56.38	0.09	0.0007	0.0307	*	K07046
1050.82	894.61	9357.83	12890.93	0.11	0.0007	0.0307	*	K08256
1817.11	2104.90	15197.96	19817.01	0.12	0.0007	0.0307	*	K16148
1312.63	3458.03	14123.43	21743.77	0.09	0.0008	0.0315	*	K00299
3870.08	4919.48	21117.16	23271.10	0.18	0.0008	0.0315	*	K00355
2327.13	2330.09	16286.37	20864.81	0.14	0.0008	0.0315	*	K00886
1903.51	2656.21	14568.57	19189.54	0.13	0.0008	0.0315	*	K00990
42.02	34.61	322.91	532.96	0.13	0.0008	0.0315	*	K01236
272.66	936.98	821.86	1338.43	0.33	0.0008	0.0315	*	K01960
4747.64	4932.50	21004.07	21853.25	0.23	0.0008	0.0315	*	K02483
2907.84	3276.64	15833.55	20021.43	0.18	0.0008	0.0315	*	K03724
42.78	37.11	327.76	534.15	0.13	0.0008	0.0315	*	K06044
1675.52	2303.43	6855.88	6507.66	0.24	0.0008	0.0315	*	K07075
4440.75	6024.48	20120.52	27736.17	0.22	0.0008	0.0315	*	K07341
2244.84	2333.97	15729.10	19756.72	0.14	0.0008	0.0315	*	K08289
2290.30	3909.87	16136.23	20724.26	0.14	0.0008	0.0315	*	K10010
90.34	356.90	743.86	1139.72	0.12	0.0008	0.0315	*	K13059
38.94	39.51	417.81	854.03	0.09	0.0008	0.0315	*	K13671
2094.37	2376.72	15438.39	19990.42	0.14	0.0008	0.0315	*	K13788
35.37	33.05	178.60	193.54	0.20	0.0008	0.0315	*	K16146
15911.62	12906.52	51231.78	41366.68	0.31	0.0009	0.0340	*	K01256
6844.63	8194.70	29062.34	24743.89	0.24	0.0009	0.0340	*	K02076
1205.24	1627.83	5828.19	6504.70	0.21	0.0009	0.0340	*	K02664
1001.47	1835.38	5463.02	6719.31	0.18	0.0009	0.0340	*	K03658
2869.81	3439.66	16334.99	18833.71	0.18	0.0009	0.0340	*	K06188
973.92	1713.56	5571.68	6590.52	0.17	0.0009	0.0340	*	K07535
234.79	771.41	505.20	441.58	0.46	0.0009	0.0340	*	K16905
3753.38	4647.51	13002.35	15505.35	0.29	0.0010	0.0367	*	K01156
29110.69	23899.24	83046.45	53757.58	0.35	0.0010	0.0367	*	K01589
13436.04	11876.32	37202.13	24048.09	0.36	0.0010	0.0367	*	K02424
1828.88	2108.22	15329.60	19943.67	0.12	0.0010	0.0367	*	K03727
33.37	31.37	261.14	424.48	0.13	0.0010	0.0367	*	K04756
26030.02	33652.24	54229.08	37324.94	0.48	0.0011	0.0382	*	K00001
7112.82	8678.47	25148.95	23967.77	0.28	0.0011	0.0382	*	K03593
1593.47	1984.93	7039.97	7963.68	0.23	0.0011	0.0382	*	K03646
312.50	1206.89	415.75	580.19	0.75	0.0011	0.0382	*	K09136
323.99	1209.65	403.76	399.54	0.80	0.0011	0.0382	*	K12339
381.04	1244.78	876.22	1100.21	0.43	0.0011	0.0382	*	K13051
117.23	363.27	846.04	1195.23	0.14	0.0011	0.0382	*	K18351
4652.74	4667.27	32807.09	41292.93	0.14	0.0011	0.0382	*	K18955
18585.73	23355.54	42764.38	34657.38	0.43	0.0011	0.0382	*	K19350

	40.46	113.18	417.29	905.19	0.10	0.0011	0.0382	*	K19355
	6203.94	7499.20	25494.18	23404.93	0.24	0.0012	0.0400	*	K01903
	3487.18	5286.49	12838.68	17828.77	0.27	0.0012	0.0400	*	K06895
	16751.07	12619.34	59030.87	51371.07	0.28	0.0012	0.0400	*	K07386
	677.57	1656.06	9829.98	16759.52	0.07	0.0012	0.0400	*	K08679
	2213.33	2462.12	16173.91	20736.09	0.14	0.0012	0.0400	*	K13288
	0.86	1.84	3.20	3.04	0.27	0.0012	0.0400	*	K13580
	429.21	1633.68	598.23	485.94	0.72	0.0012	0.0400	*	K16907
	92.69	356.78	771.75	1133.28	0.12	0.0012	0.0400	*	K18334
	16024.28	12133.18	54473.70	42193.47	0.29	0.0013	0.0425	*	K00226
	2455.87	2482.64	16553.81	21105.63	0.15	0.0013	0.0425	*	K05595
	0.07	0.23	0.91	1.45	0.07	0.0013	0.0425	*	K16319
	27.25	126.80	5.54	6.90	4.92	0.0013	0.0425	*	K19163
	914.82	1315.12	2998.40	3154.86	0.31	0.0014	0.0449	*	K01792
	2373.29	2877.13	12402.28	16309.51	0.19	0.0014	0.0449	*	K03281
	1958.72	2269.26	14147.67	18783.96	0.14	0.0014	0.0449	*	K08168
	2389.44	2963.75	14786.48	17628.96	0.16	0.0014	0.0449	*	K08728
	732.97	1264.38	9035.89	15929.01	0.08	0.0015	0.0474	*	K01181
	7601.89	7267.62	28587.46	25473.08	0.27	0.0015	0.0474	*	K08369
	336.89	499.12	2366.51	4032.87	0.14	0.0015	0.0474	*	K16922
	665.55	933.89	2274.44	2216.31	0.29	0.0016	0.0498	*	K01197
	511.69	1157.15	9035.11	16203.66	0.06	0.0016	0.0498	*	K06909
	4.61	8.15	31.20	35.59	0.15	0.0016	0.0498	*	K07445

Supplementary table 6: List of barcode primers for the Ion Torrent sequencing

SampleID	Adapter Sequence [30bp]	Barcode [10bp]	GAT + 16S Fw Primer [18bp]	Chip
IfmP01	CCATCTCATCCCTGCGTGTCTCCGACTCAG	ACGAGTGCGT	GATAYTGGGYDTAAAGNG	A
IfmP02	CCATCTCATCCCTGCGTGTCTCCGACTCAG	ACGCTCGACA	GATAYTGGGYDTAAAGNG	A
IfmP03	CCATCTCATCCCTGCGTGTCTCCGACTCAG	AGACGCACTC	GATAYTGGGYDTAAAGNG	B
IfmP04	CCATCTCATCCCTGCGTGTCTCCGACTCAG	AGCACTGTAG	GATAYTGGGYDTAAAGNG	B
IfmP05	CCATCTCATCCCTGCGTGTCTCCGACTCAG	ATCAGACACG	GATAYTGGGYDTAAAGNG	C
IfmP06	CCATCTCATCCCTGCGTGTCTCCGACTCAG	ATATCGCGAG	GATAYTGGGYDTAAAGNG	C
IfmP07	CCATCTCATCCCTGCGTGTCTCCGACTCAG	CGTGTCTCTA	GATAYTGGGYDTAAAGNG	D
IfmP08	CCATCTCATCCCTGCGTGTCTCCGACTCAG	CTCGCGTGTC	GATAYTGGGYDTAAAGNG	D
IfmP09	CCATCTCATCCCTGCGTGTCTCCGACTCAG	TAGTATCAGC	GATAYTGGGYDTAAAGNG	A
IfmP10	CCATCTCATCCCTGCGTGTCTCCGACTCAG	TCTCTATGCG	GATAYTGGGYDTAAAGNG	A
IfmP11	CCATCTCATCCCTGCGTGTCTCCGACTCAG	TGATACGTCT	GATAYTGGGYDTAAAGNG	B
IfmP12	CCATCTCATCCCTGCGTGTCTCCGACTCAG	TACTGAGCTA	GATAYTGGGYDTAAAGNG	B
IfmP13	CCATCTCATCCCTGCGTGTCTCCGACTCAG	CATAGTAGTG	GATAYTGGGYDTAAAGNG	C
IfmP14	CCATCTCATCCCTGCGTGTCTCCGACTCAG	CGAGAGATAC	GATAYTGGGYDTAAAGNG	C
IfmP15	CCATCTCATCCCTGCGTGTCTCCGACTCAG	ATACGACGTA	GATAYTGGGYDTAAAGNG	D
IfmP16	CCATCTCATCCCTGCGTGTCTCCGACTCAG	TCACGTACTA	GATAYTGGGYDTAAAGNG	D
IfmP17	CCATCTCATCCCTGCGTGTCTCCGACTCAG	CGTCTAGTAC	GATAYTGGGYDTAAAGNG	A
IfmP18	CCATCTCATCCCTGCGTGTCTCCGACTCAG	TCTACGTAGC	GATAYTGGGYDTAAAGNG	A
IfmP19	CCATCTCATCCCTGCGTGTCTCCGACTCAG	TGTACTACTC	GATAYTGGGYDTAAAGNG	B
IfmP20	CCATCTCATCCCTGCGTGTCTCCGACTCAG	ACGACTACAG	GATAYTGGGYDTAAAGNG	B
IfmP21	CCATCTCATCCCTGCGTGTCTCCGACTCAG	CGTAGACTAG	GATAYTGGGYDTAAAGNG	C
IfmP22	CCATCTCATCCCTGCGTGTCTCCGACTCAG	TACGAGTATG	GATAYTGGGYDTAAAGNG	C
IfmP23	CCATCTCATCCCTGCGTGTCTCCGACTCAG	TACTCTCGTG	GATAYTGGGYDTAAAGNG	D
IfmP24	CCATCTCATCCCTGCGTGTCTCCGACTCAG	TAGAGACGAG	GATAYTGGGYDTAAAGNG	D
IfmP25	CCATCTCATCCCTGCGTGTCTCCGACTCAG	TCGTCGCTCG	GATAYTGGGYDTAAAGNG	A
IfmP26	CCATCTCATCCCTGCGTGTCTCCGACTCAG	ACATACGCGT	GATAYTGGGYDTAAAGNG	A
IfmP27	CCATCTCATCCCTGCGTGTCTCCGACTCAG	ACGCGAGTAT	GATAYTGGGYDTAAAGNG	B
IfmP28	CCATCTCATCCCTGCGTGTCTCCGACTCAG	ACTACTATGT	GATAYTGGGYDTAAAGNG	B
IfmP29	CCATCTCATCCCTGCGTGTCTCCGACTCAG	ACTGTACAGT	GATAYTGGGYDTAAAGNG	C
IfmP30	CCATCTCATCCCTGCGTGTCTCCGACTCAG	AGACTATACT	GATAYTGGGYDTAAAGNG	C
IfmP31	CCATCTCATCCCTGCGTGTCTCCGACTCAG	AGCGTCGTCT	GATAYTGGGYDTAAAGNG	D
IfmP32	CCATCTCATCCCTGCGTGTCTCCGACTCAG	AGTACGCTAT	GATAYTGGGYDTAAAGNG	D
IfmP33	CCATCTCATCCCTGCGTGTCTCCGACTCAG	ATAGAGTACT	GATAYTGGGYDTAAAGNG	A
IfmP34	CCATCTCATCCCTGCGTGTCTCCGACTCAG	CACGCTACGT	GATAYTGGGYDTAAAGNG	A
IfmP35	CCATCTCATCCCTGCGTGTCTCCGACTCAG	CAGTAGACGT	GATAYTGGGYDTAAAGNG	B
IfmP36	CCATCTCATCCCTGCGTGTCTCCGACTCAG	CGACGTGACT	GATAYTGGGYDTAAAGNG	B
IfmP37	CCATCTCATCCCTGCGTGTCTCCGACTCAG	TACACACACT	GATAYTGGGYDTAAAGNG	C
IfmP38	CCATCTCATCCCTGCGTGTCTCCGACTCAG	TACACGTGAT	GATAYTGGGYDTAAAGNG	C

SampleID	Adapter Sequence [30bp]	Barcode [10bp]	GAT + 16S Fw Primer [18bp]	Chip
IfmK39	CCATCTCATCCCTGCGTGTCTCCGACTCAG	TACAGATCGT	GATAYTGGGYDTAAAGNG	D
IfmK40	CCATCTCATCCCTGCGTGTCTCCGACTCAG	TACGCTGTCT	GATAYTGGGYDTAAAGNG	D
IfmK41	CCATCTCATCCCTGCGTGTCTCCGACTCAG	TAGTGTAGAT	GATAYTGGGYDTAAAGNG	A
IfmK42	CCATCTCATCCCTGCGTGTCTCCGACTCAG	TCGATCACGT	GATAYTGGGYDTAAAGNG	A
IfmK43	CCATCTCATCCCTGCGTGTCTCCGACTCAG	TCGCACTAGT	GATAYTGGGYDTAAAGNG	B
IfmK44	CCATCTCATCCCTGCGTGTCTCCGACTCAG	TCTCTATGCG	GATAYTGGGYDTAAAGNG	B
IfmK45	CCATCTCATCCCTGCGTGTCTCCGACTCAG	AGCGTCGTCT	GATAYTGGGYDTAAAGNG	C
IfmK46	CCATCTCATCCCTGCGTGTCTCCGACTCAG	AGTACGCTAT	GATAYTGGGYDTAAAGNG	C
IfmK47	CCATCTCATCCCTGCGTGTCTCCGACTCAG	TGTGAGTAGT	GATAYTGGGYDTAAAGNG	D
IfmK48	CCATCTCATCCCTGCGTGTCTCCGACTCAG	AGACTATACT	GATAYTGGGYDTAAAGNG	D
IfmK49	CCATCTCATCCCTGCGTGTCTCCGACTCAG	CTCGCGTGTC	GATAYTGGGYDTAAAGNG	A
IfmK50	CCATCTCATCCCTGCGTGTCTCCGACTCAG	TACTGAGCTA	GATAYTGGGYDTAAAGNG	A
IfmK51	CCATCTCATCCCTGCGTGTCTCCGACTCAG	AGCTCACGTA	GATAYTGGGYDTAAAGNG	B
IfmK52	CCATCTCATCCCTGCGTGTCTCCGACTCAG	ACTGTACAGT	GATAYTGGGYDTAAAGNG	B
IfmK53	CCATCTCATCCCTGCGTGTCTCCGACTCAG	ATAGAGTACT	GATAYTGGGYDTAAAGNG	C
IfmK54	CCATCTCATCCCTGCGTGTCTCCGACTCAG	TCGATAGTGA	GATAYTGGGYDTAAAGNG	C
IfmK55	CCATCTCATCCCTGCGTGTCTCCGACTCAG	CGATCGTATA	GATAYTGGGYDTAAAGNG	D
IfmK56	CCATCTCATCCCTGCGTGTCTCCGACTCAG	TGTAGTGTGA	GATAYTGGGYDTAAAGNG	D
IfmK57	CCATCTCATCCCTGCGTGTCTCCGACTCAG	TCGATAGTGA	GATAYTGGGYDTAAAGNG	A
IfmK58	CCATCTCATCCCTGCGTGTCTCCGACTCAG	CGAGAGATAC	GATAYTGGGYDTAAAGNG	A
IfmK59	CCATCTCATCCCTGCGTGTCTCCGACTCAG	CGTACTCAGA	GATAYTGGGYDTAAAGNG	B
IfmK60	CCATCTCATCCCTGCGTGTCTCCGACTCAG	TGTAGTGTGA	GATAYTGGGYDTAAAGNG	B
IfmK61	CCATCTCATCCCTGCGTGTCTCCGACTCAG	TACGCTGTCT	GATAYTGGGYDTAAAGNG	C
IfmK62	CCATCTCATCCCTGCGTGTCTCCGACTCAG	TCGATCACGT	GATAYTGGGYDTAAAGNG	C
IfmK63	CCATCTCATCCCTGCGTGTCTCCGACTCAG	TAGTCGCATA	GATAYTGGGYDTAAAGNG	D
IfmP139	CCATCTCATCCCTGCGTGTCTCCGACTCAG	TACACGTGAT	GATAYTGGGYDTAAAGNG	D
